# Application of the Numerical Techniques for Modelling Fluidization Process Within Industrial Scale Boilers

**DOI:** 10.1007/s11831-016-9186-z

**Published:** 2016-08-31

**Authors:** Wojciech P. Adamczyk

**Affiliations:** Institute of Thermal Technology, Konarskiego 22C, 44-100 Gliwice, Poland

## Abstract

The numerical simulation of the large scale industrial circulating fluidized bed (CFB) boilers, working under air- and oxy-fuel combustion are presented in this paper. Moreover, two-dimensional experimental rig used for numerical model validation is described. For three-dimensional numerical simulations two industrial compact CFB boilers were selected installed in Polish Power Plants. Numerical simulations were carried out using three-dimensional model where the dense particulate transport phenomenon was simultaneously modelled with combustion process. The fluidization process was modelled using the hybrid Euler–Lagrange approach. Within the paper, readers can find information about used computational technique and a number of reference to specific work. The impact of radiative heat transfer on predicted temperature profile within the CFB boiler was investigated in presented work. Moreover, the novel model for retrieving radiative properties of gases under oxy-fuel combustion process was used. The evaluated temperature and pressure profiles during numerical simulations were compared against measured data collected during boiler air-fuel operation. Collected data was also used for validating numerical model of the oxy-fuel combustion model. Stability of the model and its sensitivity on changes of composition of the oxidizer were studied. This simulations were evaluated to check the response of the numerical model on changing the combustion conditions from air- to oxy-fuel combustion process. The comparison of the pressure and temperature profiles for all considered cases gave comparable trends in contrary to measured data.

## Introduction

Computational fluid dynamics (CFD) has reached a status of a reliable design and optimization tool in a wide range of a scientific and technological application. Robust and validated CFD models can be used as *virtual experiments*, which can help in improvement of existing processes, accelerate prototyping, optimization, and design of more efficient processes. However, inherent complexity of the modelled processes along with the presence of some empiricism in the CFD models make the verification and validation of the simulations an inevitable portion of process of the numerical modelling.

CFD solves the general transport equations with built in sub-models, allowing for modelling even very complex coupled multiphysics problems. Over years numerous commercial, in-house, and freeware CFD codes have been developed. As a rule, the software allows the users to customize material and boundary conditions, adding additional source terms into the transport equations, which makes them applicable for solving a wide range of problems. Some of the commercially available CFD codes have a closed structure. No option is provided to modify the code, which is crucial when introduction of own sub-models or stabilization of the iterative process is required. Such a closed structure excludes practically exclude such codes from applications in R&D phase. The most frequently used commercial CFD codes, as ANSYS Fluent [1], ANSYS CFX [2] or STAR-CD [3] have a semi-opened structure, where users can modify, via own subroutines, the original code. This opens the way not only to enhance the functionality of the codes, but also gives the possibility of substituting the sub-models installed in the original package. The open source codes, such as OpenFOAM [4], Dolfyn [5], and MFIX [6] have completely open structure, with the full access to the source code. While modification of a semi open code is relatively straightforward, handling the open source software requires in-depth knowledge of both the physics of the modelled phenomena and the implemented numerics. Along with these three groups of widely accessible software a number of ad hoc in-house CFD codes have been written.

**Fig. 1:**
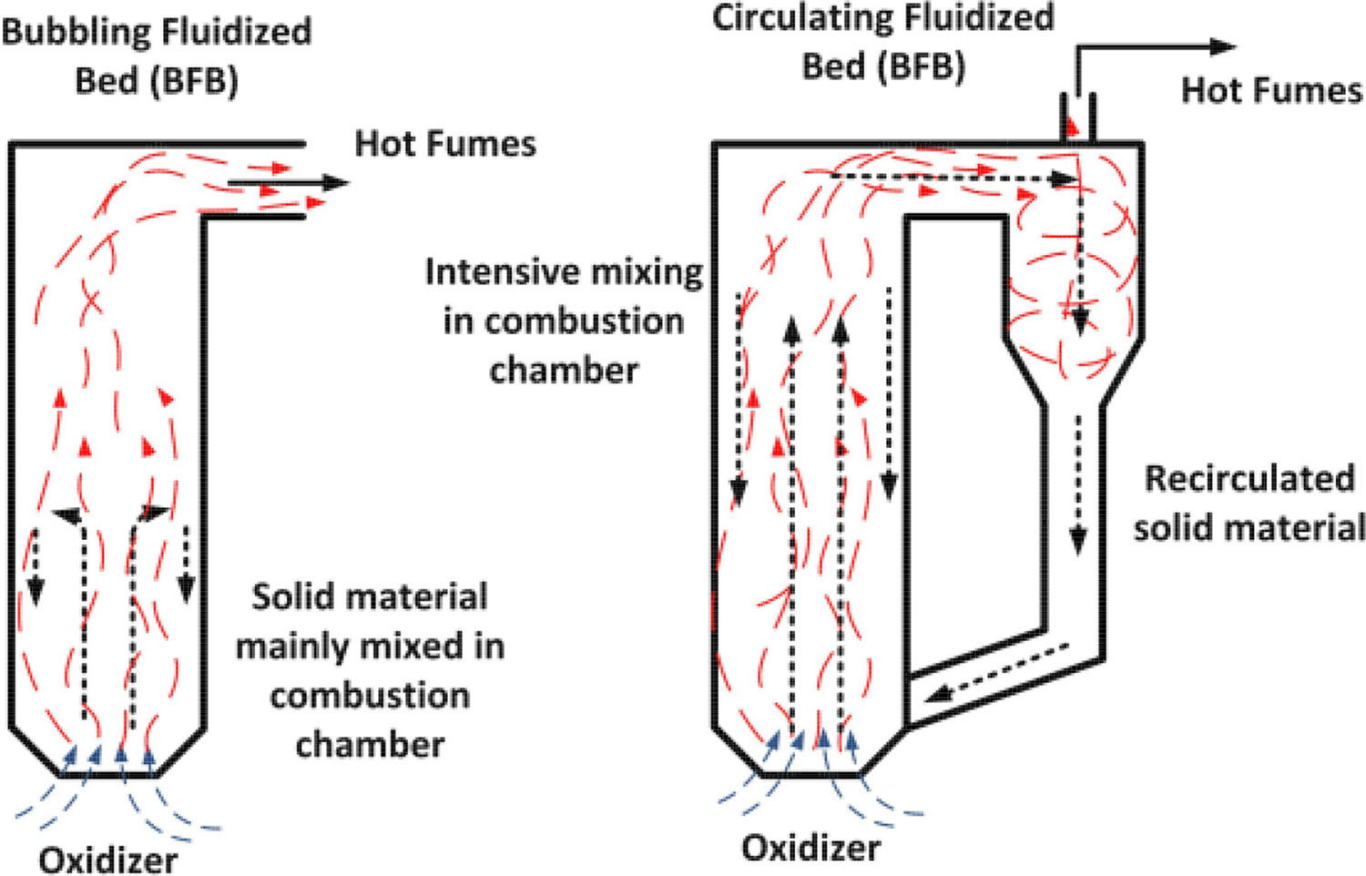
Simplified scheme of the bubbling fluidized bed (*left*) and circulating fluidized bed (*right*)

Fluidization is a multiphase phenomenon where the disperse phase (solid particles) is suspended in a moving fluid. The equilibrium of drag and gravitational forces acting on the particles makes the solid fluid mixture behaving like a homogeneous fluid. Intensive mixing in the fluidized bed enhances the mass and energy transfer, leading to compact dimensions of the equipment. Fluidization with its numerous advantages became a popular unit operation frequently applied in many branches of the process industry. Fluidized bed dryers, absorbers, catalytic chemical reactors are just a few examples of applications of this technology. Fluidized bed combustion became also a feasible alternative to the standard pulverized coal technology. The attractiveness of fluidization comes from its fuel flexibility, low cost of desulphurization and denitrification and fuel preparation. Low quality coals, biomass, sludge and wastes can burned in fluidized combustors. Low NO_x_ emission can be attributed to lower combustion temperature kept between 800 to 950 °C and $\hbox {SO}_2$ emission without introduction of complex burners or additional flue gas treatment facilities. Sulphur oxides are absorbed by calcium additives introduced to fluidized bed. In industrial application the fluidized bed boilers work typically as circulating or bubbling rectors. The scheme of such units is shown in Fig. 1.

The complexity of the physical processes in the fluidized comes from the inherent interactions between the fluid and the solid particles, as well as the particles themselves. These interactions and the presence of a wide range of space and time scales makes the modelling of the hydrodynamics of the fluidized bed a very challenging task. The presence of combustion and gasification makes the task even more demanding. To deal with the complexity of combustion in granular flow, special numerical models have to be devised. They are based on the description of the flow systems on fundamental grounds and cover the large range of scales of the involved phenomena. Based on the carried out experimental data, some of these models have been developed and validated, but this approach has been so far possible only in the context of small-scale facilities [7–10].

Such models are however not suitable for simulations of large-scale industrial boilers, which due to the limitation of the computer resources, need to be simulated using less general numerical approaches. Large scale 3D simulations of combustion in fluidized bed are practically not available in the literature. Nevertheless, model developed by Rainio [11], Hyppanen et al. [12] which has been future improved and extended by Myohanen [13] is semi-empirical model capable to model simplified real scale CFB boilers including hydrodynamics and combustion process. Some application of semi-empirical model can be found in works [14–18].

The numerical models which are capable of simulating particle transport in dense granular flow are categorized depending on how the dispersed phase is resolved. The gas and solid flow in the CFBs are frequently modeled using Euler–Euler or Euler–Lagrange approaches. The basic assumption of the Euler–Euler model is that the gas phase, as well as the disperse phase are both treated as interpenetrating continua. For both phases the set of Navier–Stokes and continuity equations are solved together with energy and turbulence closure equations. The main disadvantage of the Euler–Euler approach is that the real distribution of particles diameters cannot be modeled directly. The Euler–Euler approach assumes that all solid particles in one disperse phase are identical, which means that the particle size distribution is represented by a mean characteristic diameter and density. In order to resolve more accurately the particle size distribution, several dispersed phases have to be modeled, which is computationally expensive. In the course of combustion, the diameters of the particles change, introducing additional complexity associated with the varying mass of the solid phases.

This difficulties can be partially circumvented by applying the population balance method [19, 20] where the particle size distribution is represented by set of moments in the dispersed phase. Additional difficulties are connected with the necessity of preforming transient calculations with the time step sufficiently small to capture the fast movements of solid phase and to achieve stable calculation process. The simulation are performed until a pseudo steady state flow is achieved and performing time averaging.Typically, the time steps are in the order of 1 ms which makes those simulation time consuming. Euler–Euler technique bases on an non-physical assumption of treating the particles as a continuous phase. This adds some complexity in realistic description of the mass, energy transfer between phases and, more significantly, makes the description of chemical reactions a fairly complex matter.

Applying the Euler–Lagrange model the particle size distribution is resolved in natural way. Here the disperse phase particles are traced as they travel in the moving fluid. However, this approach is usually dedicated to flows with relatively low concentration of dispersed phase, without taking into account the interactions between particles. More sophisticated Euler–Lagrange models like discrete element method (DEM) give possibility to simulate particle interactions employing hard- or soft-sphere collision model. However, this approach is numerical expensive which excludes its application to large-scale industrial installations.

The optimal technique to deal with the dense granular transport in a reasonable short time including combustion process seems to be the hybrid Euler–Lagrange. This CFD model incorporates advantages of Euler–Euler and Euler–Lagrange approaches, taking into account particle interactions, dependencies between phases and combustion processes at a relatively low cost. However, the applicability of the hybrid Euler–Lagrange approach for predicting granular flow structures and combustion phenomena has not been investigated in both small- and large-scale CFB boilers. The research underlying this thesis concentrates on this aspect of combustion in CFB processes. The main difficulties when implementing this approach are not due to the new formulations of the technique, but the proper selection of submodels, implementation of the extended submodels for controlling behavior of the granular phase, introducing user function for reducing numerical cost of running complex simulations, and achievement of convergence in the large scale simulations.

Nowadays, a lot of effort is oriented on reduction emission, mainly $\hbox {CO}_2$ during coal combustion process. The current legislation penalizes the emission of $\hbox {CO}_2$, treated as the main reason for the greenhouse effect. A lot of efforts have been devoted to the decarbonization of the coal based power generation processes. A standard approach is to absorb the $\hbox {CO}_2$ from the combustion gases by amine and desorption the $\hbox {CO}_2$ by heating the resulting solution. The investment and running costs of the absorption installation is, however, very high, which significantly reduces the gross efficiency of the power plant. Another decarbonization technology is the oxy-combustion whose idea is to facilitate the $\hbox {CO}_2$ capture [21] from flue gases containing practically pure $\hbox {CO}_2$. In this process the oxidizer is a mixture of $\hbox {CO}_2$ and oxygen. While the former species is obtained by recirculation of the flue gases, oxygen is produced in an Air Separation Unit (ASU). As a result, the flue gases contain practically only $\hbox {CO}_2$, which drastically reduces the cost of $\hbox {CO}_2$ capture. The price to pay is the energy needed to separate air into oxygen and nitrogen. This technology can be applied to conventional pulverized coal and fluidized bed boilers [22, 23]. The change from air to oxy-combustion exerts a significant impact on all transport phenomena taking place in the combustion chamber. The reduction of the volumetric flow rate of flue gases, change of heat capacity and transport properties (heat and mass diffusivity) are main reasons for this.

This paper presents application of the hybrid Euler–Lagrange approach for modelling particle transport and combustion phenomena within the laboratory scale experimental-rig and industrial scale CFB boilers. Readers, can find wide description of available methods which can be used for modelling such complex multifluid process in various applications. Paper consists valuable experimental and measure data, as well as geometrical models which can be used for validating other computational technique. The application of the hybrid Euler–Lagrange technique, with extended submodels based on user defined functions implemented into the solution procedure and purposed methodology which can be used for solving dense granular flows within industrial scale boiler operated under air and oxy-fuel combustion process has not been earlier presented in literature. All developed functions and calculation strategy ensure stable operation of the numerical model with high accuracy, robustness and stability. Developed model at the Institute of Thermal Technology has been already used in several authors application [24–27]. Is worth to say hear that model is constantly developed to deal with erosion process, sulphur capture, chemical looping and it is also intensively validated against laboratory scale test rigs.

### Current Status of the Research

When the fluidized bed is modelled numerically, different space and time scales have to be considered. References [14, 28] analyse different scales, described in terms of modelling gas–solid dynamic and mixing are distinguished. The space and time scales have been divided into micro-, meso- and macro-scales. The micro-scale defines the molecular level of the particles, the meso-scale is related to small flow structures like clusters, whereas the macro-scale stands for large scale and mixing processes. Typically, when fluidization process is modelled the meso- and macro-scales models like the Euler–Euler and Euler–Lagrange are used. The most popular and widely used approach, applied for modelling fluidization process is the multi-fluid Euler–Euler approach [29–31] which is classified as measo- or macro-space scale models [14]. In this approach, both dispersed and continuous phases are treated as interpenetrating continua. This approach has been derived based on the assumption that the solid phase can be treated simultaneously as a continuous medium with representative properties similar to the fluid [29]. The governing equations of the Euler–Euler model, as well as the closure terms have evolved during the many years of the applications. The first governing equations for two-phase systems were presented in Anderson and Jackson [32]. A similar approach has been described in the work of Ishii [33]. In Ref. [34] these two models were compared. The conclusion was that the model developed by Anderson and Jackson is more appropriate for gas–solid systems while the Ishii’s model better reflects the behaviour of the gas–liquid flows. The application of multifluid model for predicting of particle transport phenomena requires additional closure models. Those models are used for predicting particle collisions in the dispersed phase, fluid–solid interaction any many other physical relations between the involved phases. Based on the theory of dense gases [35] the kinetic theory of granular flow (KTGF) has been developed and first applied to granular flows by Jenkin and Savage [36] and Lun et al. [37] for smooth, spherical and nearly elastic particles. The KTGF model was further developed to take into account the dissipation of kinetic fluctuation energy in the granular medium during non-ideal particle–particle collisions and due to the interaction of solid phase with surrounding gases in dense gas–solid fluidized beds [30, 38, 39]. The Euler–Euler technique has been applied in simulations of small scale CFB units [9, 40–45] and spouted beds [46, 47].

The modelling process of the particle transport in the fluidized bed should accurately predict the wide range of a particle size distribution (PSD) and the chemical processes during combustion. In the case of the combustion and gasification processes the particles size changes dynamically in the course of the process, affecting the fluidized bed hydrodynamics [48]. The changes in the diameters during such processes can span several orders of magnitudes, indicating that the PSD has to be accurately reproduced. When Euler–Euler approach is used, the real distribution of the particle diameters cannot be tracked directly at low cost. In order to deal with the PSD each of the characteristic diameters has to be modelled by a separate solid phase. This is accomplished by extending the approach to a multi-dispersed system, where each dispersed phase is represents by the same material properties (density, specific heat, etc.) but of different diameter. In the work of Syamlal et al. [49] the applicability of this approach is presented. In order to faithfully represent the PSD many dispersed phases are required. This expensive approach can be avoided by resorting to the population balance method [20, 50]. Many approaches exist to solve these equations. Historically, the method of moments (MOM) has been one of the first technique uses for this purpose. The advantages of this technique is that the overall PSD can be represented by a small number of equations for the unknown moments (i.e., usually 4–6). From this point of view, the implementation of the MOM technique in CFD codes is advantageous. The difficulty in the implementation of the MOM lies in expressing the transport equations in terms of the moments themselves (closure problem). To circumvent this disadvantage McGrow [51] developed the quadrature methods of moments (QMOM) which approximates the unclosed terms by quadrature. The weights and abscissas of the quadrature can be determined from lower-order moments [52] by applying the product-difference (PD) algorithm proposed by Gordon [53]. Marchisio et al. [54] has implemented the QMOM approach in the commercial computational fluid dynamics code [1] for modelling particle aggregation and breakage. Over the years the QMOM technique has been extensively validated for many applications [55–57]. The disadvantage of the QMOM when applied to predict the PSD in fluidized bed is, that each particle size calculated from moments, has the same phase-average velocity. To overcome this problem the direct quadrature method of moments (DQMOM) has been formulated and successfully validated [58]. In this approach the weights and abscissas are calculated directly by solving transport equations for this quadrature weights. In DQMOM formulation each node of the quadrature can be represented by separate dispersed phase, with its own velocity [19, 59, 60]. Some application of DQMOM can be found in [61].

A straightforward approach where the discrete particle are modelled directly has a number of conceptual advantages. However, the direct numerical solution, or Lattice Boltzmann methods [62] are limited to a small-scale problems. The discrete models applied to dense dispersed phase in the fluidized bed boiler can accurately predict particles and particle fluid interaction and the PSD in dispersed phase. This can be achieved by using discrete particle or discrete element method (DP or DEM) [63]. This two approaches describe the particle interaction using the hard-sphere and soft-sphere collision models. The hard-sphere model, assumes that interactions between particles are instantaneous [64]. The particle collision effect is identified between each of the particle pairs, which is numerical expensive. The soft-sphere model uses the Hertzian contact theory, which models slight overlapping of particles during contact [65]. Due to the intensive calculations, the usability of the DEM is limited to small scale problems. Some applications of the hard-sphere DEM for modelling of particle transport in small-scale fluidized beds can be found in [66, 67], whereas Tsuji et al. [68] used soft-sphere collision model for simulating of bubbling fluidized bed, employing 4.5 millions of particles. Even with the increasing computer power DEM is numerically very expensive and cannot be applied to realistic engineering problems. In order to speed up the numerical simulation without losing accuracy, a hybrid Euler–Lagrange Multiphase Particle-In-Cell (MP-PIC) approach has been developed by Andrews and O’Rourke [69]. This method was later enhanced by Patankar [70, 71] and Snider [72–74]. Using the MP-PIC approach particles are tracked in Lagrangian frame of reference, whereas the interactions between particles are calculated within the Eulerian grid using continua models for the solid-phase stress. In this approach instead of tracking individual particles, groups of particles (clouds), represented by a fixed number of identical particles with the same physical proprieties are traced. The particle collisions in the dispersed phase are not resolved explicitly, but the effect of particle collisions is accounted for in an calculated solid-phase stress in computational cell. This collision model has been recently improved by O’Rourke et al. [75, 76]. The MP-PIC method is numerically less expensive than other Euler–Lagrange approaches, thus it can be applied to handle larger scale systems, simulating particles with different sizes and materials proprieties. This model has been successfully used in commercial closed CFD code CPFD Baracuda [77]. The disadvantages of the close code is the inability of modification of the implemented models and introduction of own sub-models. At this moment the published MP-PIC applications including particle transport, combustion and gasification are still limited to small- and middle-scale units [74, 78–80]. Currently the modified version of the MP-PIC model has been implemented in the commercial Ansys FLUENT 14.0 CFD code [1] as a hybrid Euler–Lagrange Dense Discrete Phase Model (DDPM). This semi open package gives a possibility of including user defined functions (UDFs) for particle transport and particle interaction phenomena. With the full control of those processes the code allows to improve and enhance the implemented sub-models. In the present work those functions have been used for calculating pressure drop in the fluidized bed, solid recirculation procedure for the Euler–Euler and hybrid Euler–Lagrange approaches, and modification of drag model for modelling particle transport in large scale CFB boiler using Eulerian technique.

### Fluidization

**Fig. 2:**
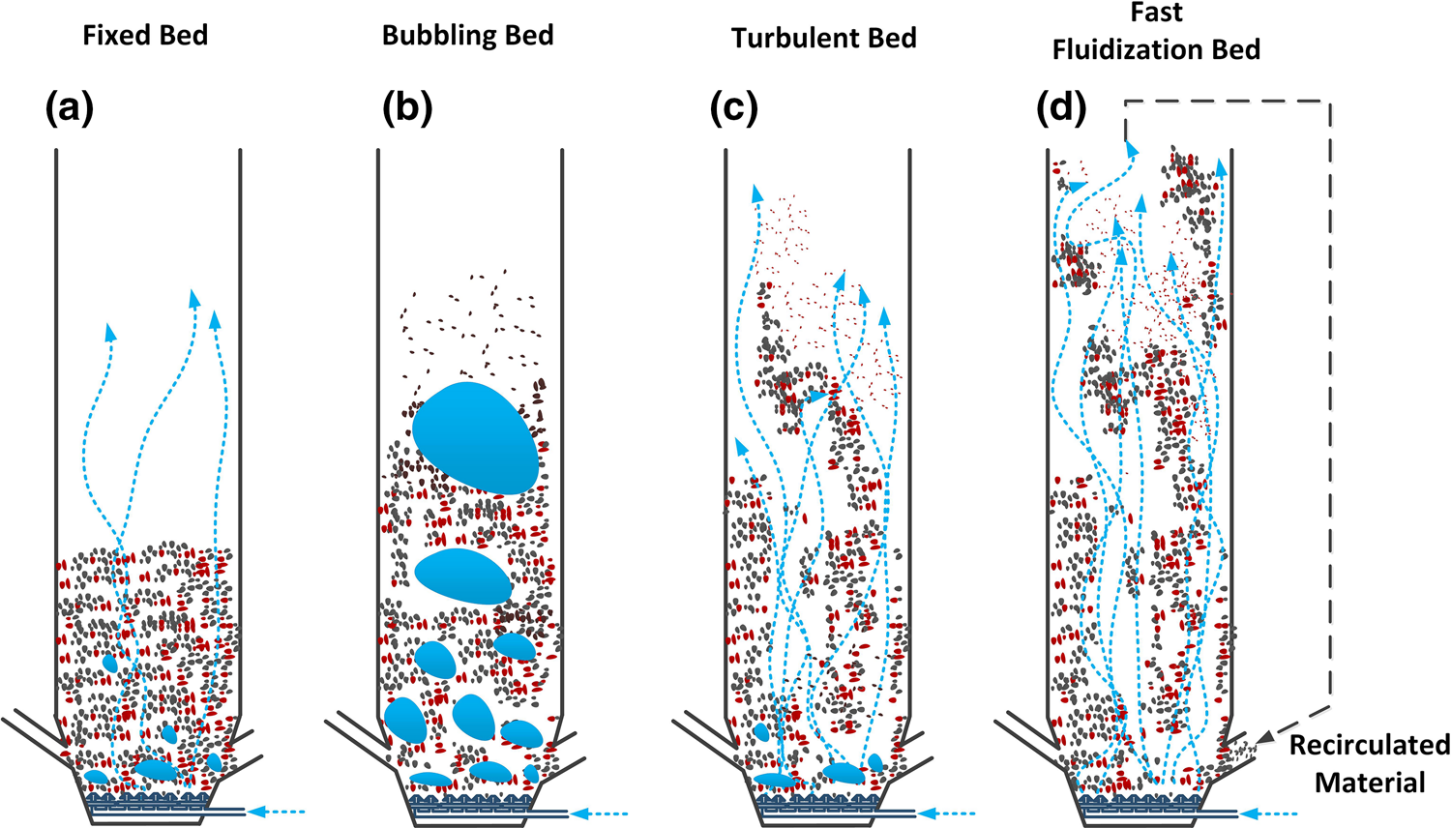
Steps of the fluidization process

The fluidization is a process where due to the gas flow through the boiler, the solid material starts mixing and moving over the boiler chamber. At the beginning of the process, the bed of particles rest on the distributor (often a porous solid). At low flow rates, gas flows though the distributor penetrates the layer of the particles that behave as a porous medium, which is shown in Fig. 2a. When the frictional (*drag*) forces between carrier gas and particles are large enough to compensate the gravity and other forces, the particles start to move in the bed. In this situation the velocity of the gas which flows through the bed achieves the minimum fluidization velocity $u_{{\mathrm {mf}}}$, see Fig. 2b. When the gas velocity increases beyond that of minimum fluidization velocity the bed porosity increases and the gas bubbles are formed Fig. 2c. Further increase of the gas velocity to the so called *terminal velocity* $u_{{\mathrm {tf}}}$, moves the small particles from the upper part of the bed into the the combustion chamber. When the gas velocity increases above the terminal velocity $u_{{\mathrm {tf}}}$, the bubbles are substituted by turbulent motion of particles (see Fig. 2d). Additional increase of the gas velocity transforms the turbulent bed into the fast fluidized bed. Fluidized particles are removed from the reactor and via the solid separator recirculated back to the bed, see Fig. 2e.

#### Flow Regimes in Fluidized Bed Boilers

**Fig. 3:**
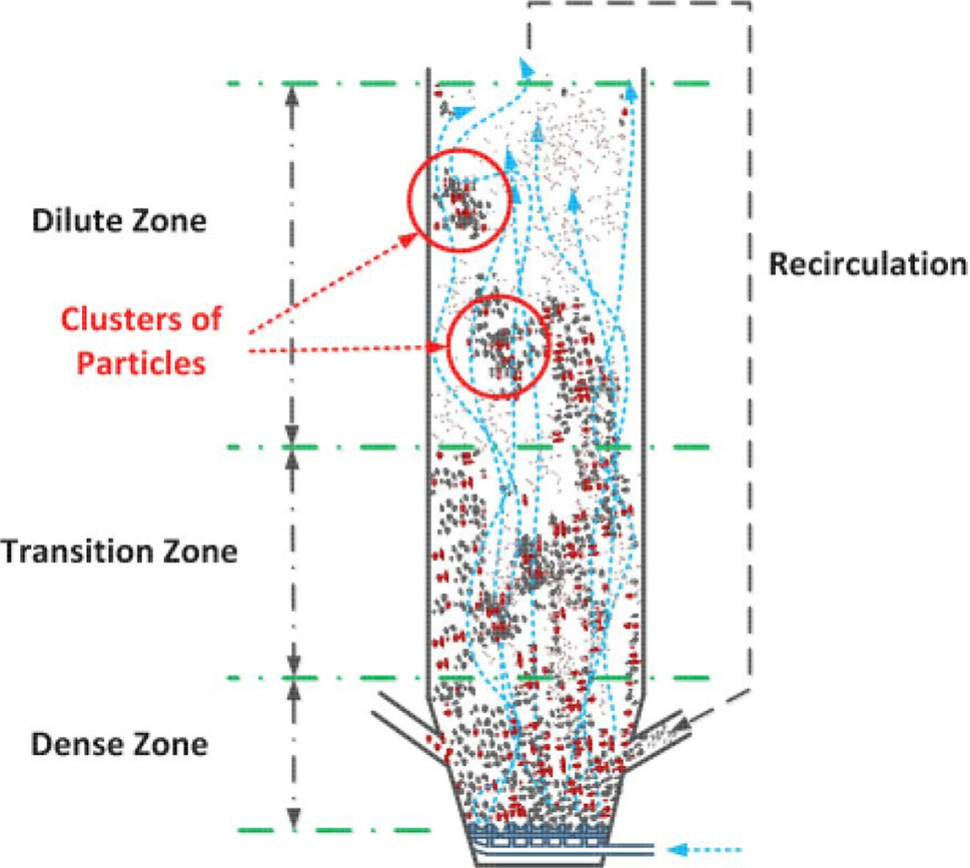
Flow regimes for dilute, transition and dense particle flows

**Fig. 4:**
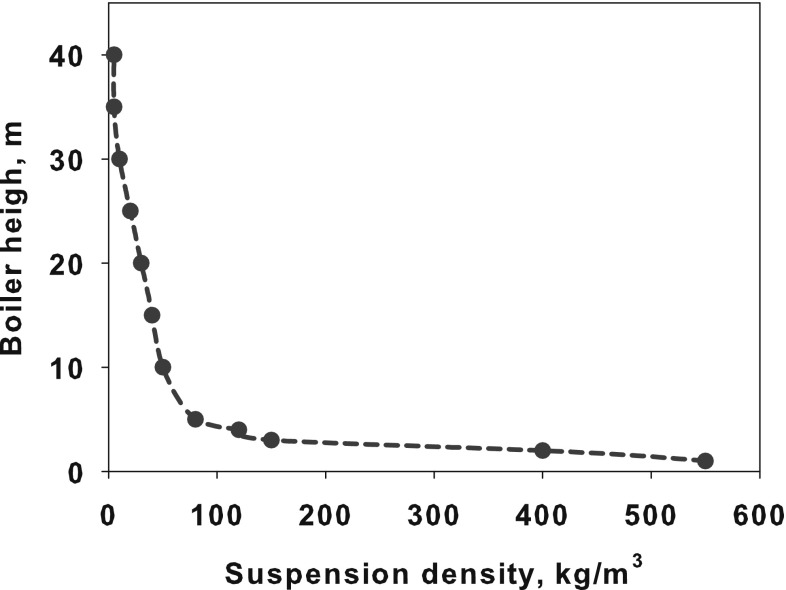
Distribution of the suspension density $\rho _s(1-\varepsilon _f)$ in fluidized bed boiler [82]

As shown in Fig. 3 three characteristic zones in fluidized bed boiler can be distinguished, namely the dense, transition and dilute ones. With increasing distance from the gas distributor the particle concentration decreases, generating characteristic profile of the mass distribution in the fluidized bed boiler shown in Fig. 4. In the dense zone the fast fluidized bed is developed. Because of the high concentration of the solid material in this region, particles can gather into clusters. In such clusters the interactions within the solid phase are mainly caused by the friction forces. Here the instantaneous particle collisions process is less important as the distance between the particles is relatively low. However, solid stresses which take into account friction and kinetic transport can attain very high values, causing expansion of solid clusters to transition region. In the transition zone the friction force is no longer dominating. The distance between particles increases and the particles friction phenomena is replaced by the particle collision. Due to the large distance between particles in the dilute zone, the interaction between the particles is dominated by the kinetic transport.

The fluidization process as well as the ranges of the fluidization zones depends on the particle size, density and concentration. Small particles with relatively low density can easily be fluidized and entrained from the bed to the transition and next to dilute zones. With increasing mass (pressure drop) of the bed the value of the minimum fluidization velocity responsible for starting fluidization process also increases. The influence of the particles diameter and density on the particle mixing is described by Geldart [81].

### Circulating Fluidized Bed

**Fig. 5:**
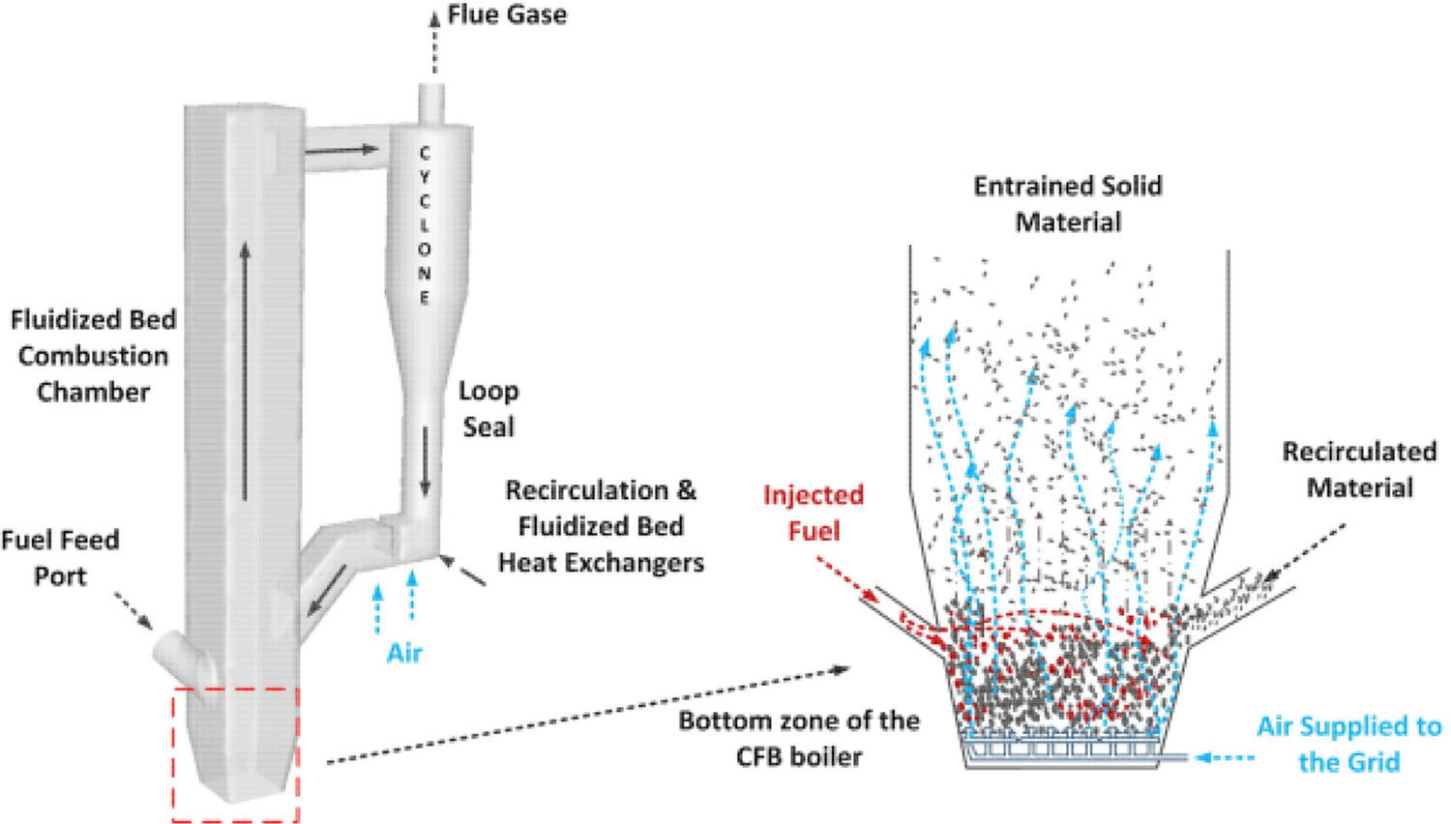
Scheme of a circulating fluidized bed boiler used in the power generation sector

The circulating fluidized bed (CFB) boilers are by far the most popular fluidized bed technology in combustion. The temperatures in CFB are below the ash softening temperature for nearly all fuels. As a result, the furnace design is independent of ash characteristics. This allows a boiler to handle a wide range of fuels. The overall residence time of fuel particles is in CFB boilers much higher, than in the standard PC and BFB boilers, ensuring complete fuel combustion and increasing overall boiler efficiency. The CFB boilers are suitable for power generation from waste and biomass, bituminous and lignite coal as well as co-combustion of these fuels. The advantages of CFB boilers are especially pronounced for units whose power exceeds 350 MWe. Figure 5 shows the scheme of a typical CFB boiler. Typical configuration of the CFB includes combustion chamber, hot solid separators (cyclone, compact) and loop seal. Under typical operation conditions the gas velocity is maintained over the entrainment velocity, which depends on the fuel type and boiler construction. Beyond this velocity the bed material becomes entrained from the turbulent bed and the particles are distributed throughout the furnace with a gradually decreasing density from the bottom to the top of the furnace, as shown in Fig. 4.

The design of the CFB furnace involves fuel conversion by controlling several parameters including: operating temperature, gas velocity, gas/solids residence times, and solid circulation rates.

Primary oxidizer stream is admitted at the base of the furnace via the oxidizer distributor (separator). This configuration generates highly turbulent bed composed of fuel, oxidizer, inert and adsorbent. To maintain good solid fluidization, the cross section area of the furnace at the base of the boiler is often smaller than the upper part. The entrained material from the bed is constantly transported to the upper part of the boiler, where it is recirculated back via the solid separators. Large particles are carried by the flue gases only to certain height of the furnace. When the resultant of the drag and other forces acting on the particle are smaller than the gravitational force, the particles slid down on the boiler walls. The size of particles in the lower part of the boiler is reduced due to erosion, cracking of particles, devolatilization and combustion.

**Fig. 6:**
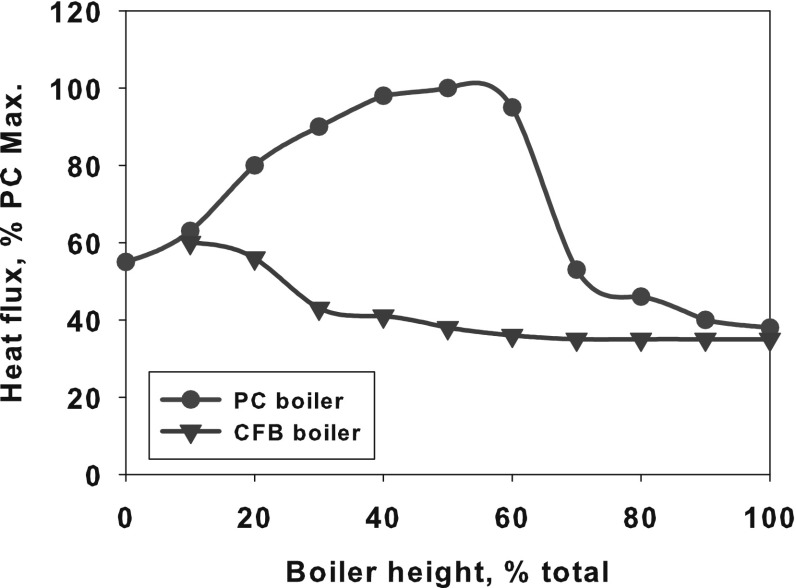
Heat flux to the boiler walls for both PC and CFB boilers [83]

The enclosure walls of the CFB combustion chamber are shielded by water tubes and a layer of refractory material similarly as in the BFB boiler. In the CFB boiler the combustion temperature is relatively low and uniform, kept in range 800–950 °C. The heat flux in the CFB boiler are lower than in the PC furnace, which is illustrated in Fig. 6. The relatively low and uniform heat fluxes to the boiler walls (see Fig. 6) protects the evaporator tubes from dry-out processes. This reduces the boiler capital costs as internal water tubes rifling need not be installed. Moreover, due to lower mass rates, the operating costs of pumping/circulation of water is lower than in the PC boilers.

### The Particle Hydrodynamics and Heat Transfer

The hydrodynamic interactions between the gas and solid (dispersed) phases are responsible for the complexity of the gas–solid flows. The hydrodynamic forces can be decomposed into several groups. The gravitational force is balanced by the fluid drag and buoyancy forces which act in opposite direction. When those forces are in pseudo-equilibrium state, the particle is suspended in carried gas. The fluidized bed behaves than as if it were a fluid, and can be described by mathematical tools developed to deal with the continuous phase [84]:

- the static pressure at any height in the vessel is approximately equal to the weight of the solid bed per unit of cross-sectional area above that level where the pressure acts,
- an object which has higher density than the density of the bed will sink, while the lighter objects will float on a surface following Archimedes principle,
- the solids from the bed may be drained like a liquid through an orifice at the bottom or on the side of the container,
- the solid flow-stream can be treated similar as a water jet,
- the bed surface maintains a horizontal level, independent how the container is tilted, moreover the bed assumes the shape of the vessel,
- for well mixed particles the bed maintains a nearly uniform temperature when heated.

The differences between solid and fluid flow can be noticed in an hour-glass and in a U-tube pipe. In hour-glass which is used as the device for controlling time, the particle flow rate through the orifice in the bottom is constant and independent of particle bed high. This is opposite to fluid where the flow rate depends on the hydrostatic head. In the U-tube when it is filled by the fluid both arms contain the same amount of water, whereas when instead of fluid the sand will be used, only one arm of the U-tube is filled.

The pressure drop across the bed results from the drag forces acting on the particles immersed in moving fluid. The pressure drop per unit height of a packed bed $\varDelta p/H$ with uniformly sized particles can be correlated using Ergun equation [85]

1 $$\frac{\varDelta p}{H} = \underbrace{\left[ 150\frac{(1-\varepsilon _f)^2}{\varepsilon ^3_f}\frac{\mu _f}{(k_vd_p)^2} + 1.75\frac{\rho _f(1-\varepsilon _f)u_f}{\varepsilon ^3_fd_pk_v}\right] }_{K_{fs}} u_f$$

where term $K_{fs}$ is defined as the inter-phase exchange coefficient between phases, $\varepsilon _f$ is the void fraction of the bed, $d_p$ is the diameter of the particles, $u_f$ is the superficial velocity of the gaseous phase, i.e., velocity that the fluid would have through the empty tube at the same volumetric flow rate, *H* is the bed height and $k_v$ is the particle sphericity which for ideal sphere is equal to $\pi /6$. Increasing the pressure drop in bed, the superficial gas velocity $u_f$ tends to a critical value known as the minimum fluidization velocity $u_{{\mathrm {mf}}}$. For bed at rest the pressure drop can be defined as [30]

2 $$\varDelta p = H(1-\varepsilon _f)(\rho _p-\rho _f)g$$

where *H* is the height of the bed, $\rho _p$ is the particle density, and *g* is the gravity. The minimum fluid velocity $u_{{\mathrm {mf}}}$ at which the bed starts to fluidize, can be calculated by solving simultaneously Eqs. 1 and 2 assuming $u_f=u_{{\mathrm {mf}}}$.

**Fig. 7:**
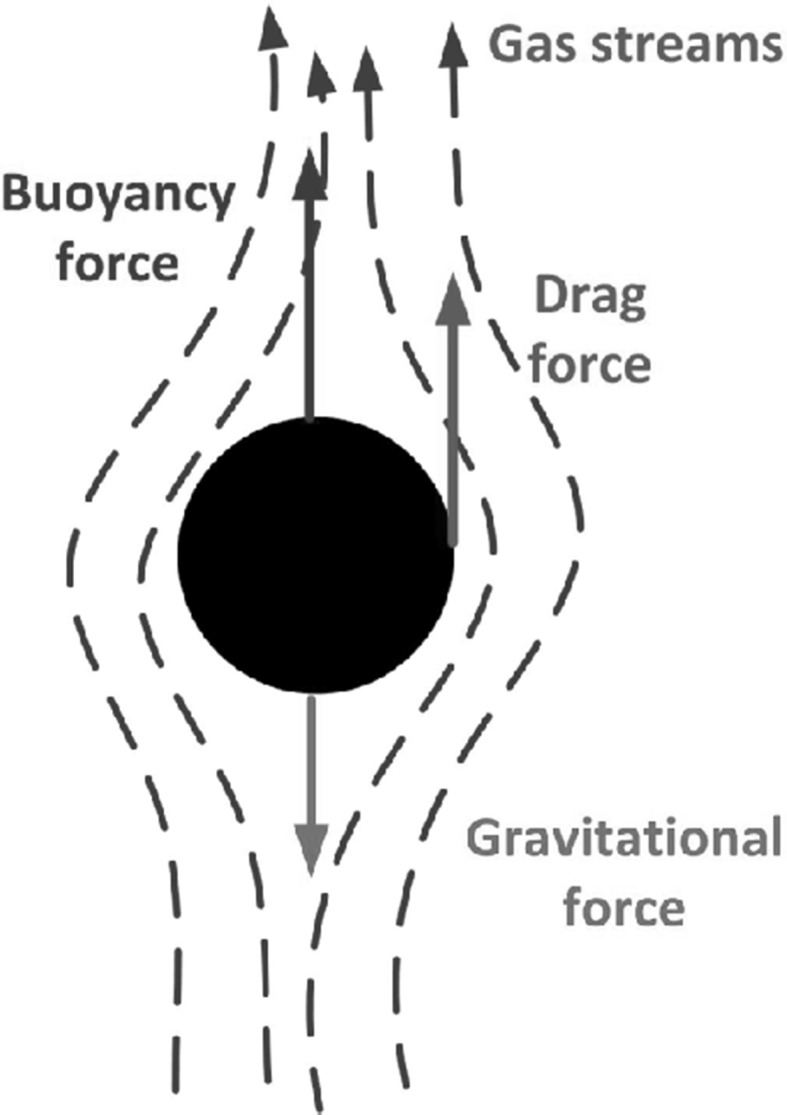
Forces which acts on a particle within gas stream [84]

Other important velocities which are responsible for particle transport phenomena in fluidized bed are the terminal velocity $u_{{\mathrm {tf}}}$ and relative velocity $u_{{\mathrm {r}}}$. The terminal velocity can be calculated from particle force balance equation (3) under assumption, that the gas velocity $u_f$ is equal to zero, and particle velocity $u_p$ is equal to terminal velocity $u_{{\mathrm {tf}}}$. Particle balance equation (3) has been derived for a single particle movement under gravitational, buoyancy and drag forces, as it is presented in Fig. 7

3 $$\underbrace{m_pg}_{{\mathrm {Gravity}}}=\underbrace{m_p\frac{\rho _fg}{\rho _p}}_{{\mathrm {Buoyancy}}} + \underbrace{C_{\mathrm {D}}\frac{\pi \left( u_f-u_p\right) ^2\rho _f}{8}d_p^2}_{{\mathrm {Drag}}}$$

where $\left( u_f-u_p\right)$ is defined as the relative velocity (slip velocity, $u_{\mathrm {r}}$) which represents particle resistance against falling, when the gas and particle move upwards, $C_{\mathrm {D}}$ is the drag coefficient. Here it should be stressed that the forces presented in Fig. 7 and particle balance equation (3) are right only for single particle carried by the fluid.

Other parameter which influences the particle transport in gaseous phase is the non-dimensional Stokes number defined as

4 $${\mathrm {St}}=\frac{\tau _{\mathrm {F}}}{\tau _{\mathrm {R}}}$$

where $\tau _{\mathrm {F}}$ stands for the characteristic time of the flow field and $\tau _{\mathrm {R}}$ is the response time of the particle. The response time is defined as the time required for changing the particle velocity by carrier gas. For very low values of Stokes number near to zero the particle has ample time to response to gas velocity. In such situation the particle and gas velocity are nearly equal. This future is used by the particle image velocimetry (PIV) [86], however when Stokes number is much higher than one, the particle velocity is unaffected by the gas.

**Fig. 8:**
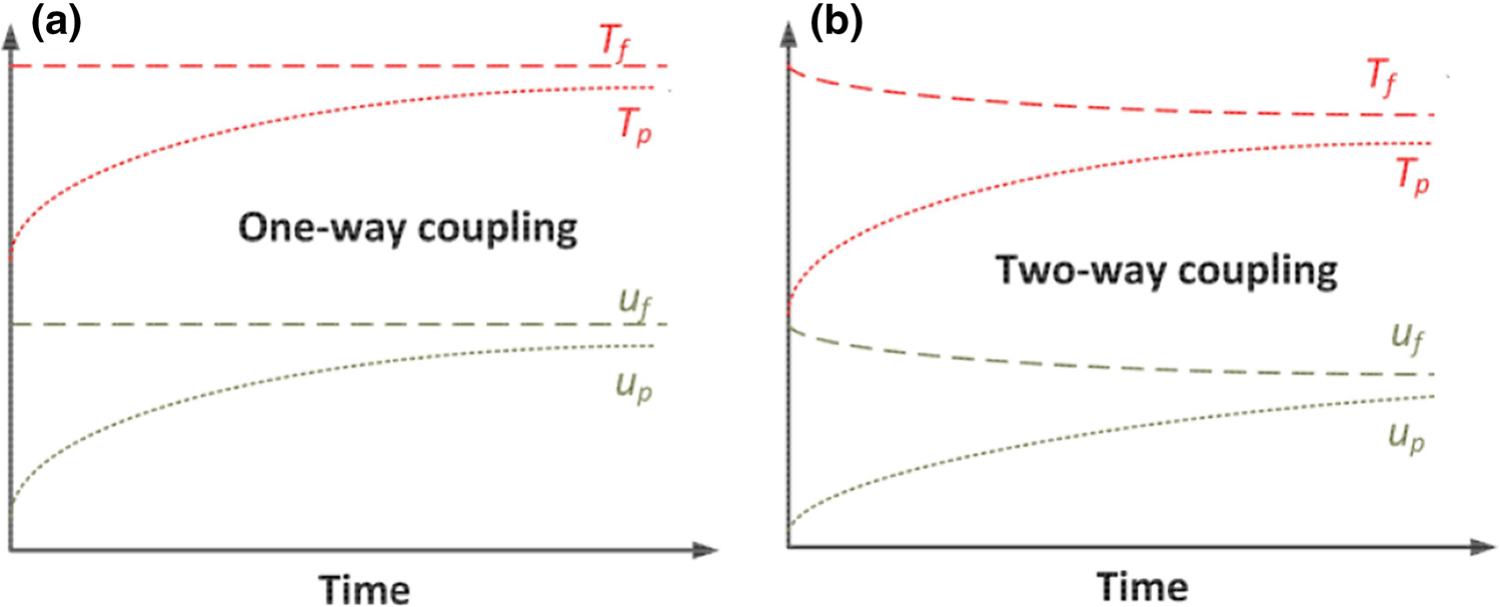
Illustration of one- and two-way coupling during particle heating process [63]

In multiphase flows where solid phase is relatively diluted $(1-\varepsilon _f)<0.1$ one- and two-way coupling between phases come into play [63]. For such cases the interactions between particles can be omitted. In one-way coupling the gaseous phase affects the particle motion, while there is no reverse effect of the particle movement on the fluid. Should both direction of influence be accounted for, the coupling is termed *two way coupling*. The coupling between the phases is even more important, when the heat transfer between phases has to be taken into account. Changes of the particle temperature affect the evaluated velocity field of the carrier gas, as shown in Fig. 8. In one-way coupling the changes of particle temperature do not affect gas temperature and velocity field of the gaseous phase as shown in Fig. 8a. When two-way coupling is accounted for, the heat transfer between phases influences both the velocity and temperature fields of the gaseous phase (see Fig. 8b). When combustion and gasification processes are considered, the two-way coupling between phases cannot be neglected. When the solid volume fraction exceeds 10 %, interactions between particles in dispersed phase also have to be taken into account. The interactions within dispersed phase are described in more detail in Sect. 2.2.1.

The changes of particle velocity is described by the particle acceleration equation (6) taken into account the gravitational and buoyancy forces

5 $$\frac{{\mathrm {d}}{\mathbf {u}}_p}{{\mathrm {d}}t}=\frac{18 \mu _f}{\rho _p d_p^2}\frac{C_{\mathrm {D}}{\mathrm {Re}}}{24}\left( {{\mathbf {u}}}_f-{\mathbf {u}}_p \right) + \frac{{\mathbf {g}}\left( \rho _p - \rho _f\right) }{\rho _p}$$

where $\rho _p$ is the density of the solid phase, ${\mathbf {u}}_f$ is the velocity vector of the gaseous phase interpolated form computational cell where particle is actually located, ${\mathbf {u}}_p$ stands for the exact particle velocity. For known particle velocity, its position ${\mathbf {x}}$ can be evaluated using simple relation

6 $$\frac{{\mathrm {d}}{\mathbf {x}}_p}{{\mathrm {d}}t}={\mathbf {u}}_p$$

**Fig. 9:**
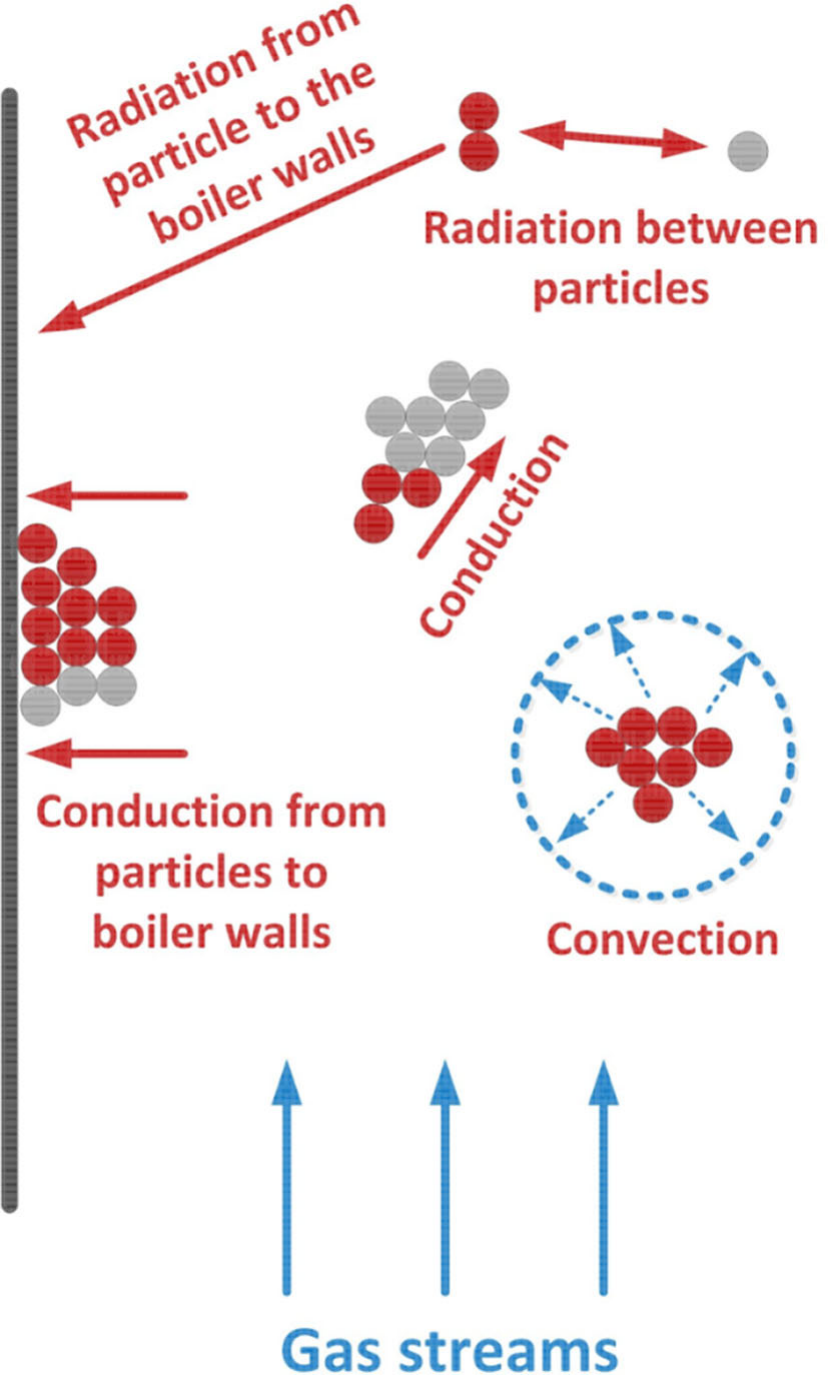
Heat transfer in the fluidized bed

Along with the gravitational, drag and buoyancy forces the particles can be exposed to other forces like virtual mass, thermophoretic, Brownian, Basset, Magnus and Saffman’s lift forces. The conditions under which these forces need to be included in the analysis are described in Refs. [45, 63, 87]. Within the fluidized boiler several sections responsible for heat transfer processes can be distinguished. Most of the combustion process takes place in the combustion chamber of the fluidized bed furnace. Released heat due to chemical reaction is transferred to the boiler walls or, it is exchanged between particles by conduction, convection or radiation. This is schematically shown in Fig. 9. Combusted particles together with a hot inert material from combustion chamber are directed to solid separators, which can work as external solid super-heaters [88] for steam generation process. High rate of heat transfer can be observed in the vicinity of the distributor, where fast mixing process takes place. In this region large temperature difference between bed and gases occur.

The hot gases from solid separators are passed to the convective or back-pass section of the boiler where economizers are installed. The mathematical model of the combustion process for single particle is comprehensively described in Sect. 3. Additional information also can be find in work of Basu [84].

## Mathematical Model of Transport Phenomena in Fluidized Bed

### Standard Euler–Euler Approach

The Euler–Euler approach for describing particle transport in isothermal conditions (*cold flow*) without mass transfer between phases uses a set of transport equations including the conservation of mass and momentum. Equations 7 and 8 are the continuity transport equation for gaseous and solid phases, respectively, whereas Eqs. 9 and 10 define the momentum change between the fluid and solid phases, respectively [1, 14]. These transport equations are presented in instantaneous form without terms responsible for mass transfer between phases. Interested readers are referred to following Refs. [29, 30, 32] for detailed derivation of the governing equations.

7 $$\begin{aligned} \frac{\partial }{\partial t}\left( \varepsilon _{f}\rho _{f}\right) +\nabla \cdot \left( \varepsilon _f\rho _f{\mathbf {u}}_f\right)= 0 \end{aligned}$$

8 $$\frac{\partial }{\partial t}\left( \varepsilon _{s}\rho _{s}\right) +\nabla \cdot \left( \varepsilon _s\rho _s{\mathbf {u}}_s\right)= 0$$

9 $$\frac{\partial }{\partial t}\left( \varepsilon _{f}\rho _{f}{\mathbf {u}}_f\right) + \nabla \cdot \left( \varepsilon _f\rho _f{\mathbf {u}}_f{\mathbf {u}}_f\right)= -\varepsilon _f\nabla p + \nabla \cdot \tau _f + \varepsilon _f\rho _f {\mathbf {g}} + \sum ^{N_s}_q\left[ K_{qf}\left( {\mathbf {u}}_f - {\mathbf {u}}_q\right) \right]$$

10 $$\frac{\partial }{\partial t}\left( \varepsilon _{s}\rho _{s}{\mathbf {u}}_s\right) + \nabla \cdot \left( \varepsilon _s\rho _s{\mathbf {u}}_s{\mathbf {u}}_s\right)= -\varepsilon _s\nabla p + \nabla \cdot \sigma _s + \varepsilon _s\rho _s {\mathbf {{g}}} + K_{fs}\left( {\mathbf {u}}_f - {\mathbf {u}}_s\right) + \sum ^{N_s-1}_q\left[ K_{qs}\left( {\mathbf {u}}_q - {\mathbf {u}}_s\right) \right]$$

where ${\mathbf {g}}$ is the gravity, the subscripts *f* and *s* denote the gaseous and solid phases respectively, $\varepsilon$ denotes the phase volume fraction, $\rho$ is the density, ${\mathbf {u}}$ defines the velocity vector, *p* is the pressure of gaseous phase, and *K* represents the interphase exchange coefficients between phases, the subscript *q* stands for the *q*-th solid phase and $N_s$ is the total number of solid phases. The $\tau _f$ is the fluid stress tensor which represents viscous forces in fluid phase which can be calculated as [1]

11 $$\tau _f= \varepsilon _f \mu _f \left( \nabla {\mathbf {u}}_f+\nabla {\mathbf {u}}^{T}_{f}\right) + \varepsilon _f\left( \lambda _f - \frac{2}{3} \mu _f \right) \nabla \cdot {\mathbf {u}}_f \bar{{\mathbf {I}}}$$

where $\lambda _f$ is the bulk viscosity of fluid phase, $\bar{{\mathbf {I}}}$ is the unit tensor, $\mu _f$ represents the fluid dynamic viscosity. For incompressible flow the term $\nabla \cdot {\mathbf {u}}_f$ in Eq. 11 is equal to zero, as well as the bulk viscosity in this equation. The set of multiphase transport equations is solved by the CFD code in Reynolds averaged form [63] in order to predict field variables quantifying by the average volume fraction occupied by each phase.

The phases volume fractions $\varepsilon _f$ and $\varepsilon _{s}$ are determined using averaging procedures like phase volume averaging described in [49, 63, 89] or ensemble averaging [40, 89–91]. To solve a set of multifluid transport equations the closure terms, which define interaction between phases, have to be defined. Detailed description of the definition of the closure terms can be found in Sect. 2.2.1.

Using Euler–Euler approach the question of diameter distribution of the granular phase requires additional attention. To account for this important question, the population of the particles is divided into a finite number of fractions of representative diameter. When using this approach, the continuity equations should be solved for every fraction. This leads to numerically intensive computations. To circumvent this problem the population balance technique has been introduced. Several versions of this technique have been developed. The description of these group of methods can be found elsewhere [20, 59, 61]. Due to their numerical instability and long execution times these methods have been excluded from further tests.

### Hybrid Euler–Lagrange Model

An alternative to the Euler–Euler or populations balance model technique of dealing with diameter distribution of the particle population is the hybrid Euler–Lagrange technique. This approach can be described by several mathematical models. Nowadays, two of them are mainly used, the Dense Discrete Phase Model (DDPM) [1, 92] and the Multiphase Particle in Cell (MP-PIC) model [69, 70, 72]. The DDPM approach has common roots with the MP-PIC technique where four-way coupling procedure [14] is used to take into account the interaction between phases, as well as the interactions between particles in the dispersed phase. The interactions between particles are accounted for by resorting to the kinetic theory of granular flow (KTGF) described in [30, 37]. This theory takes into account the mutual interaction between particles using reasoning originally developed for kinetic molecular theory of dense gases [35]. In the continuous phase, the influence of the particles movement and the energy transfer between them and the fluid carrier is accounted for by additional terms included in the conservation equations. The dispersed phase is considered both as a continuum and as a discrete phase where particles are tracked in Lagrangian frame of reference. Particle properties are mapped to and from an Eulerian grid. The data transfer between the Eulerian grid and particle position is carried out by interpolation operators [69, 73]. The interpolation method is utilized for resolving of the inter-particle stress, which is difficult to calculate for each particle in the dense flow. It is assumed that the gradient of solid stress can be calculated on the Eulerian grid and then its value interpolated to the discrete particle position [72].

When the hybrid Euler–Lagrange DDPM approach is used for simulating the reacting flow inside the fluidized bed boiler, the transport equations of mass, momentum, energy, species and also the turbulence model have to be solved. To take into account the density fluctuations during the combustion process, caused by significant variations of both temperature and flue gases concentration, the Favre averaging procedure [93] has to be used for the aforementioned transport equations. The standard RANS approach can be applied for flows characterized by low Mach number treating gases as incompressible aside density fluctuations. In Favre averaging procedure the density-weighted mean velocity takes the form

12 $${\tilde{{\mathbf {u}}}_f}=\frac{\overline{\rho _f {\mathbf {u}}_f}}{\bar{\rho }_f}$$

where ${\mathbf {u}}_f$ is the velocity vector, $\bar{\rho }_f$ is the time averaged density. Using Favre averaging the instantaneous velocity is derived from simple equation

13 $${\mathbf {u}}_f=\tilde{{\mathbf {u}}}_f+{\mathbf {u}}_f^{''}$$

where ${{\mathbf {u}}_f}$ stands for mean time velocity and ${\mathbf {u}}^{''}_f$ represents turbulent velocity fluctuation and hold also the information on density fluctuations. The mass, momentum and energy equations for the gaseous phase in instantaneous form are defined as [1, 76, 92]

14 $$\frac{\partial }{\partial t}\left( \varepsilon _{f}\rho _{f}\right) + \nabla \cdot \left( \varepsilon _f{\rho }_f{{\mathbf {u}}}_f \right)= S_{{\mathrm {mass}}}$$

15 $$\frac{\partial }{\partial t}\left( \varepsilon _{f}{\rho }_{f}{{\mathbf {u}}}_f\right) + \nabla \cdot \left( \varepsilon _f{\rho }_f{{\mathbf {u}}}_f{{\mathbf {u}}}_f\right)= -\varepsilon _f\nabla {p} + \nabla \cdot {\tau }_f + +\varepsilon _f{\rho }_f {\mathbf {g}} + K_{DPM}\left( {{\mathbf {u}}}_s-{{\mathbf {u}}}_f \right) + S_{{\mathrm {mom}}}$$

16 $$\frac{\partial }{\partial t}\left( \varepsilon _{f} {{\rho }}_{f} {h}_f\right) +\nabla \cdot \left( \varepsilon _f{{\rho }}_f{{\mathbf {u}}}_f{h}_f \right)= \varepsilon _f \frac{\partial p}{\partial t} + \tau _f : \nabla {\mathbf {u}}_f - \nabla \cdot {\mathbf {q}}_f - \nabla \cdot \left[ \sum _{k=1}^{m} \varepsilon _f h_{f,k} {\mathbf {J}}_k \right] + S_{f,{\mathrm {rad}}}+ S_{f,{\mathrm {rec}}}+S_{{\mathrm {en}}}$$

17 $$\frac{\partial }{\partial t}\left( \varepsilon _{f}{{\rho }}_{f} {Y}_{f,k}\right) +\nabla \cdot \left( \varepsilon _f{{\rho }}_f{{\mathbf {u}}}_f {Y}_{f,k}\right)= \nabla \cdot \varepsilon _f {\mathbf {J}}_k + \varepsilon _f R_{f,k} +{\mathcal {R}} +S_{\mathrm {sp}}$$

where the subscripts *f* and *s* denote the fluid and solid phase respectively, *k* is the species index, *h* is the enthalpy, $Y_{f,k}$ stands for the mass fraction of species *k* in fluid phase, $K_{{\mathrm {DPM}}}$ is the drag coefficient calculated for the average value of the solid volume fraction in a numerical cell, ${\mathrm {Sc}}_{f,k}$ is the Schmidt number, *m* stands for the number of species in resolved flow, ${\mathbf {J}}$ is the diffusion flux of species *k*, and ${\mathbf {q}}$ is the heat flux. The source term $S_{f,{\mathrm {rad}}}$ defines the contribution to the energy equation due to the radiation. The source term $S_{f,{\mathrm {rec}}}$ stands for the amount of energy released from chemical reactions. The $R_{f,k}$ represents the net rate of production of homogeneous species *k*, whereas ${\mathcal {R}}$ is the heterogenous reaction rate in case of modelling surface combustion. The $S_{{\mathrm {mass}}}$, $S_{{\mathrm {mom}}}$, $S_{{\mathrm {en}}}$ and $S_{{\mathrm {sp}}}$ are sources due to exchange of mass, momentum energy and species between the continuous phases and particles in discrete phase, respectively. The energy source $S_{{\mathrm {en}}}$ includes the enthalpy transfer due to convection, chemical reactions and radiation from discrete phase. The momentum source term $S_{{\mathrm {mom}}}$ determines the change of momentum in the gaseous phase due to particles movement.

The DDPM approach does not solve motion equation for individual particles, which is also the case of MP-PIC technique. The solver tracks groups of particles called parcels. Each parcel contains several particles of the same mass, velocity, position, composition, etc. The number of individual particles contained in the injected parcel can readily be calculated from

18 $$n_p=\frac{\dot{m}_{{\mathrm {parcel}}}\varDelta t}{m_{p}}$$

where $\varDelta t$ is the time step in transient calculation, $\dot{m}_{{\mathrm {parcel}}}$ mass flow rate of single parcel and $m_p$ is the mass of individual particle evaluated based on the particle diameter and density. The particle equation of motion which equates the particle inertia with the forces acting on a particle, reads then

19 $$\frac{{\mathrm {d}}{\mathbf {u}}_p}{{\mathrm {d}}t}=F_{\mathrm {D}}({{\mathbf {u}}}_f-{\mathbf {u}}_p) + \frac{{\mathbf {g}}(\rho _p-{\rho }_f)}{\rho _p} - \frac{\nabla {p}}{\rho _p} - \frac{\nabla \cdot \sigma _s}{\rho _p}$$

where subscript *p* denotes the particle data (in one dispersed phase several materials with different physical properties can be tracked). $\sigma _s$ is the granular stress tensor which represents particles interactions calculated based on the KTGF [30, 35, 37] in the Eulerian grid. $\rho _p$ is the particle material density, $F_{\mathrm {D}}({{\mathbf {u}}}_f-{\mathbf {u}}_s)$ is the particle acceleration due to the drag. The drag coefficient $F_{\mathrm {D}}$ is calculated using the same drag model as this used for predicting the drag coefficient $K_{{\mathrm {DPM}}}$. The term $-\nabla p/\rho _p$ defines the particle acceleration due to the pressure difference at the particle location.

Based on the calculated particle velocity a new position of the particle is calculated as

20 $$\frac{{\mathrm {d}}{\mathbf {x}}_p}{{\mathrm {d}}t}={\mathbf {u}}_p$$

After obtaining the particle position, the solid volume fraction in a given numerical cell can be calculated as

21 $$\varepsilon _s=\frac{\sum ^{N_{\mathrm {parcels}}}_{i=1}V_{p,i}n_{{\mathrm {p}},i}}{V_{{\mathrm {cell}}}}$$

where $V_p$ is the considered particle volume, $V_{{\mathrm {cell}}}$ is the numerical cell volume, $k_{\mathrm {v}}$ defines the particle sphericity and $d_p$ represents the particle diameter. The calculated solid volume fraction is assigned to Eulerian grid where the void fraction can be determined as $\varepsilon _f=1-\varepsilon _{s}$. The particle velocity and position obtained by solving Eqs. 19 and 20, strongly depends upon the evaluated solid stresses $\sigma _s$ (see Eq. 22) in the Eulerian grid. In order to calculate the solid stress tensor several closure terms have to be calculated.

#### Closure Terms

In order to calculate the solid stress tensor $\sigma _s$, which accounts for interactions between particles within solid phase, several closure terms have to be defined. Closure terms are used in a description of the granular pressure, solid bulk viscosity and shear viscosities. The solid stress tensor can be defined as [49]

22 $$\sigma _s = -p_s\bar{{\mathbf {I}}}+ \varepsilon _s \mu _s \left( \nabla {\mathbf {u}}_s+\nabla {\mathbf {u}}^{T}_{s}\right) + \varepsilon _s \left( \lambda _s - \frac{2}{3} \mu _s \right) \nabla \cdot {\mathbf {u}}_s \bar{{\mathbf {I}}}$$

where $\lambda _s$ is the bulk viscosity, $\bar{{\mathbf {I}}}$ is the unit tensor, $p_s$ is the granular pressure, $\mu _s$ represents the solid dynamic viscosity, and ${\mathbf {u}}_s$ stands for the average velocity vector of the solid phase acquired at the particle location. Further granular phase modelling requires mathematical description of the dynamic viscosity, bulk viscosity and the solid pressure. The flow regime occurring in the system, understood as the solid–solid momentum exchange mechanism occurring at different solid volume fractions, determines the dispersed phase modelling. Particle collisions and kinetic transport are of a great importance for low solid volume fractions. When the volume fraction for the particulate matter is high, the particle collisions are no longer instantaneous and therefore friction between particles and kinetic transport controls the transport. Thus, dynamic viscosity of the solid phase can be expressed as a superposition of three terms

23 $$\mu _s=\mu _{s,{\mathrm {kin}}}+\mu _{s,{\mathrm {col}}}+\mu _{s,{\mathrm {fric}}}$$

in which $\mu _{s,{\mathrm {col}}}$, $\mu _{s,{\mathrm {kin}}}$, and $\mu _{s,{\mathrm {fric}}}$ represent the viscosity due to collisions, kinetic transport and friction, respectively. Several models derived from the KTGF for calculating the granular viscosity can be found in literature. In this work, the correlations representing the viscosities due to kinetic transport by [30] and collisions by [49] are applied

24 $$\mu _{s,{\mathrm {kin}}}= \frac{\varepsilon _s\rho _s d_s \sqrt{\varTheta \pi }}{6(2-e_{ss})} \left[ 1+\frac{2}{5}(1+e_{ss})(3e_{ss}-1)\varepsilon _s g_{0,ss} \right]$$

25 $$\mu _{s,{\mathrm {col}}}= \frac{4}{5}\varepsilon ^2_s \rho _s d_s g_{0,ss} (1+e_{ss}) \left( \frac{\varTheta }{\pi }\right) ^{1/2}$$

where $e_{ss}$ represents the inelastic nature of particle collisions, known as the restitution coefficient, and $\varTheta$ is the granular temperature. The probability of particle collisions $g_{0,ss}$ can be calculated as [94]

26 $$g_{0,ss}=\left[ 1- \left( \frac{\varepsilon _s}{\varepsilon ^*_{s}} \right) ^{1/3} \right] ^{-1}$$

where $\varepsilon ^*_{s}$ stands for the maximum packing limit of particles. The radial distribution function tends to infinity when a distance between particles approaches the value of particle diameter and tends to unity when the distance is increasing. The restitution coefficient $e_{ss}$ represents the fraction of energy locally dissipated due to particle–particle or particle–wall collisions. When a plastic collision occurs (total collision energy is dissipated) the restitution coefficient is equal to zero, whereas for an elastic collision (total collision energy is conserved) its value is equal to unity. In situations when the solid volume fraction exceeds the defined transition limit (friction limit) $\varepsilon ^{fr}_s$, the model dedicated for a dense regime (friction regime) is activated. In this regime the collision part of the solid viscosity is replaced by the friction viscosity $\mu _{s,{\mathrm {fric}}}$ defined as [95]

27 $$\mu _{s,{\mathrm {fric}}}=\,\frac{p_s \sin \phi }{2 \sqrt{I_{2D}}}$$

where $I_{2D}$ stands for the second invariant of the deviatoric stress tensor, $\phi$ is the angle of internal friction and $p_s$ is the granular pressure. The granular pressure, similarly to granular viscosity, is split into three terms which represent the kinetic transport, collisions and friction. The relation for the granular pressure in case of dilute flows can be written as [37]

28 $$p_{s}=\varepsilon _s \rho _s \varTheta + 2\varepsilon ^2_s \rho _s (1+e_{ss})g_{0,ss}\varTheta$$

where the first term represents the kinetic transport and the latter stands for the transport due to collisions. In dense regions, where the solid volume fraction exceed the transition volume fraction (friction limit) $\varepsilon ^{fr}_{s}$, the term representing the collision pressure is replaced by the solid friction pressure $p_{s,{\mathrm {fric}}}$ derived based on the KTGF theory [35] defined as [45]

29 $$p_{s,{\mathrm {fric}}}=10^{25}(\varepsilon _s-\varepsilon ^{fr}_s)^{10}$$

The bulk viscosity present in Eq. 22 defines the resistance of solid body to dilatation and it can be modelled as [37]

30 $$\lambda _s=\frac{4}{3}\varepsilon _s \rho _s d_s g_{0,ss} (1+e_{ss}) \sqrt{\frac{\varTheta }{\pi }}$$

One of the most important parameters, which corresponds to interactions between gas and particles, is the drag exchange coefficient *K* between phases. The relationship representing the drag coefficient is typically obtained experimentally, based on the pressure drop measurements in fluidized or settling beds. In the current work the model proposed by [30] has been used. This approach combines two closure approximations, namely the Ergun model [96], which holds for solid volume fractions exceeding 0.2 and Wen and Yu model [97] is used in regions where solid volume fraction is sharply smaller than 0.2. The Ergun and Wen and Yu model are given by Eqs. 31 and 32, respectively

31 $$K_{{\mathrm {Ergun}}}= 150\frac{ \varepsilon ^2_s \mu _f}{ \varepsilon _s d_s^2} + 1.75 \frac{\rho _f \varepsilon _s \left| {\mathbf {u}}_s-{\mathbf {u}}_f\right| }{d_s}$$

32 $$K_{{\mathrm {Wen\,and\,Yu}}}= 0.75 C_{\mathrm {D}} \frac{\varepsilon _s \varepsilon _f \rho _f \left| {\mathbf {u}}_s-{\mathbf {u}}_f\right| }{d_s} \varepsilon _{f}^{-2.65}$$

where $C_{\mathrm {D}}$ is the drag coefficient defined as

33 $$C_D=\frac{24}{\varepsilon _f {\mathrm {Re}}}\left[ 1+0.15(\varepsilon _f {\mathrm {Re}})^{0.687}\right]$$

A closer inspection of Eqs. 31 and 32 shows that there is a discontinuity at $\varepsilon _s = 0.2$ [98]. To overcome this difficulty, a blending function is frequently employed, making the model smooth. Thus the drag coefficient model can be written as [98]

34 $$K=\phi (\varepsilon _s) K_{{\mathrm {Ergun}}}+\left( 1-\phi (\varepsilon _s)\right) K_{{\mathrm {Wen\,and\,Yu}}}$$

where $\phi (\varepsilon _s)$ is the blending function defined as

35 $$\phi (\varepsilon _s)=0.5+\frac{\arctan \left[ 262.5(\varepsilon _s-0.2)\right] }{\pi }$$

When two or more additional dispersed phases are modeled additional drag coefficient has to be calculated between those phases. This has been accomplished by applying interphase symmetrical drag coefficient initially proposed by Syamlal et al. [49] and defined as

36 $$K_{{\mathrm {sym}},qs}=\frac{3 (1+e_{qs})\left( \frac{\pi }{2}+C_{{\mathrm {fric}},qs}\frac{\pi ^2}{8} \varepsilon _s\rho _s\varepsilon _q\rho _q\left( d_q+d_s\right) ^2g_{0,qs}\right) }{2\pi \left( \rho _qd_q^3+\rho _sd_s^3 \right) }\left| {\mathbf {u}}_q-{\mathbf {u}}_s\right|$$

where *q* denotes additional dispersed phase, $e_{qs}$ is the coefficient of restitution, $C_{{\mathrm {fric}},qs}$ defines the friction between the *q* th and *s* th solid phase particles and $g_{0,qs}$ is the radial distribution function.

#### Granular Temperature

The granular temperature is described as mean square value of the the random particle velocity fluctuations about the mean flow velocity [31] and can be seen as some kind of turbulent kinetic energy or energy of the solid velocity fluctuations. This quantity cannot be explicitly measured as no granular temperature thermometer is known. An exhaustive overview of various aspects of granular temperature can be found in review paper [99]. The granular temperature $\varTheta$ in constitutive equations for the granular pressure, viscosity and drag force represents thus the particles velocity fluctuation *C*

37 $$\varTheta =\frac{1}{3}\left\langle C^2_x+C^2_y+C^2_z\right\rangle$$

The granular temperature defined for each solid phase *s* is defined as

38 $$\frac{3}{2}\left[ \frac{\partial }{\partial t} (\varepsilon _s \rho _s \varTheta ) + \nabla \cdot (\varepsilon _s \rho _s \varTheta {\mathbf {u}}_s) \right] = \nabla \cdot (k_{\varTheta }\nabla \varTheta ) - \nabla p_s+\tau _s:\nabla {\mathbf {u}}_s-\gamma _s+\varphi _{fs}$$

where $\gamma _s$ is a dissipative term which represents the rate of energy dissipation within the solid phase due to collisions between particles, $\tau _s:\nabla \bar{u}_s$ represents the fluctuating energy caused by the forces acting between particles (viscous dissipation), $\nabla \cdot (k_{\varTheta }\nabla \varTheta )$ stands for the diffusion, $\varphi _{fs}$ is the exchange term which represents the kinetic energy transfer between phases [49], $k_{\varTheta }$ is the conductivity of the granular temperature and $p_s$ is the granular pressure. Due to a high instability of Eq. 38 it is typically replaced by an algebraic formulation described in [34] and [49]. Algebraic equation for granular temperature has been derived based on assumptions that the granular kinetic energy does not change significantly in time and is dissipated locally, hence the terms representing the convection and diffusion can be neglected [29, 49]. The differential equation for the granular temperature is then simplified to

39 $$-\nabla p_s+\tau _s:\nabla {\mathbf {u}}_s-\gamma _s+\varphi _{fs}=0$$

The energy dissipation due to particle collisions $\gamma _s$ is usually calculated using expression proposed by Lun at al. [37]

40 $$\gamma _s=\frac{12(1-e^2_{ss})g_{0,ss}}{d_s\sqrt{\pi }}\rho _s \varTheta ^{3/2} \varepsilon ^2_s$$

When the restitution coefficient $e_{ss}$ in Eq. 40 tends to unity, the dissipation of kinetic energy within the solid phase becomes negligible $\gamma _s\rightarrow 0$.

## Modeling Air- and Oxy-Combustion Process

The generalized model used during coal combustion processes, including heating, evaporation, devolatilization, char oxidation are described in this section. To simulate numerically combustion process of coal particles several assumptions have to be introduced [87]:

- the particle temperature is uniform,
- the coal particle is composed of ash, moisture, volatile and char,
- ash is treated as inert material,
- the coal particle are spherical and have homogeneous physical and chemical properties,
- several processes cannot take place simultaneously, one of the process has to be finished to occur the next one,
- char combustion starts once all the volatile matters have evolved, however in reality they can overlap,
- the composition of volatiles which contain hydrocarbon, nitrogen, oxygen, sulfur, carbon is defined as input for devolatilization reaction,
- moisture loss is controlled by heat transfer to the particle and vapor diffusion form coal particle to the gaseous phase.

When the combustion process is taken in to account, the variation of mass and temperature of the particles have to be taken into accounted. This is accounted for by solving particle mass and energy balance equations. The mass changes of coal particle are described by an equation

41 $$\frac{{\mathrm {d}}{m}_p}{{\mathrm {d}}t}=\frac{{\mathrm {d}}m_{{\mathrm {char}}}}{{\mathrm {d}}t}+\frac{{\mathrm {d}} m_{{\mathrm {vol}}}}{{\mathrm {d}}t}+\frac{{\mathrm {d}} m_{\mathrm {w}}}{{\mathrm {d}}t}$$

where $m_{{\mathrm {char}}}$, $m_{{\mathrm {vol}}}$ and $m_{{\mathrm {w}}}$ are the mass of the char, volatiles and water in combustible particle respectively. During combustion the amount of ash material in the coal particle remains constant. The heat transfer between surrounding gases and particle can be described by solving the particle energy balance specified as

42 $$m_p c_p \frac{{\mathrm {d}}{T}_p}{{\mathrm {d}}t}=A_{{\mathrm {ext}}}h\left( {T_f}-T_p\right) + Q_c+ A_{{\mathrm {ext}}}\epsilon \sigma \left( {\varPhi }^4-T^4_p\right)$$

where $c_p$, $T_p$ stands for the particles heat capacity and temperature, *h* is the heat transfer coefficient to particle calculated using the correlation of Ranz and Marshall on the Nusselt number [100], $A_{{\mathrm {ext}}}$ represents the particle surface area, $\epsilon$ is the emissivity of the particle surface, $Q_c$ stands for the changes of energy due to the evaporation and surface combustion processes, whereas the $\varPhi$ is the incident radiation temperature calculated as

43 $${\varPhi }=\left( \frac{G}{4\sigma }\right) ^{1/4} \rightarrow G=\int ^{4\pi }_{\varOmega =0}I{\mathrm {d}}\varOmega$$

where *I* stands for incoming radiation intensity, $\varOmega$ is the solid angle.

In first stage of the combustion process the injected particle is heated up to a defined evaporation temperature. Over the evaporation temperature the evaporation process proceed until moisture is removed from the particle. After evaporation the particle is again heated to devolatilization temperature, above which the gaseous fraction are released from the particle to the continuous phase, where the volatile matters are combusted. After devolatilization the char combustion process starts. When the entire char in the particle is consumed, the heating process is again activated and remaining hot ash is used as the inert material for heat transfer processes.

### Particle Heating Process

If the temperature of the injected particle is lower than the specified evaporation temperature, it is exposed to heating process by hot surrounding gases. The heating process is governed by particle energy equation 42, excluding energy exchange term $Q_c$. During this process the particle diameter and density remains constant. The particle heating process can also be activated between other coal combustion processes.

### Evaporation Process

The time of evaporation process depends on the amount of water which is carried with coal particle. For fuels with high moisture fraction, accuracy of prediction of the evaporation may strongly influence the overall accuracy of the simulation of coal combustion. For a typical hard coal the water fraction is relatively small (3–10 %) and in PC boilers it is almost totally removed in coal mills (transferred to hot oxidizer). For lignites, especially when they used to fire fluidized bed boilers, the evaporation can play an important role, as the moisture content is often of the order of 30 %.

The evaporation process couples heat and mass transfer. The mass transfer can be calculated taking into account only diffusion process. The change of mass of the particle during evaporation is than defined as

44 $$\frac{{\mathrm {d}}m_p}{{\mathrm {d}}t}=-N_{{\mathrm {{H_{2}}O}}} A_{\mathrm {ext}}M_{{\mathrm {{H_{2}}O}}}$$

where $M_{{\mathrm {{H_{2}}O}}}$ is the mass weight of water vapour, $N_{{\mathrm {{H_{2}}O}}}$ is the molar flux of water vapour calculated as

45 $$N_{{\mathrm {{H_{2}}O}}} = k_c \left( C_{{\mathrm {{H_{2}}O}},p} - C_{{\mathrm {{H_{2}}O}},f} \right)$$

where $k_c$ is the mass transfer coefficient calculated from Sherwood number Eq. 46, $C_{{\mathrm {H_2O}},p}$ is the concentration of the vapour at the particle surface and $C_{{\mathrm {H_2O}},f}$ defines the concentration of vapour in the bulk gas. The Sherwood number is defined as

46 $${\mathrm {Sh}}=\frac{k_c d_p}{D_{{\mathrm {H_2O}},m}}=2.0+0.6{\mathrm {Re}}^{0.5}{\mathrm {Sc}}^{0.33}$$

where $D_{{\mathrm {H_2O}},m}$ is the diffusion coefficient of the vapour in the gaseous phase, ${\mathrm {Sc}}$ is the Schmidt number. The concentration of the water vapour on the particle surface and in the core of the gas are evaluated from

47 $$C_{{\mathrm {H_2O}},p}= \frac{p_{\mathrm {sat}}(T_p)}{RT_p}$$

48 $$C_{{\mathrm {H_2O}},f}= X_{{\mathrm {H_2O}}}\frac{p}{RT_f}$$

where $p_{\mathrm {sat}}(T_p)$ is the saturation pressure for particle temperature $T_p$, *R* is the universal gas constant (8314.12 J/kmol K), $X_{{\mathrm {H_2O}}}$ is the mole fraction of water vapour in the core of the gas, $T_f$ is the temperature of surrounding gases and *p* is the absolute pressure. Chan et al. [101] observed that the duration of the evaporation in both air- and oxy-combustion regime is similar. In the course of evaporation the energy balance equation of the particle (42) includes evaporation source $Q_c$ which stands for the amount of energy absorbed from the surrounding gases to evaporate the moisture from the particle. This quantity is defined as

49 $$Q_c=\frac{{\mathrm {d}}m_p}{{\mathrm {d}}t}h_{{\mathrm {fg}}}$$

where $h_{{\mathrm {fg}}}$ is the enthalpy of evaporation defined as

50 $$h_{{\mathrm {fg}}}(T_p) = h_{{\mathrm {bp}}}+\int ^{T_{p,{\mathrm {bp}}}}_{T_p}c_{\mathrm {w}}{\mathrm {d}}T - \int ^{T_{p,{\mathrm {bp}}}}_{T_p}c_{\mathrm {g}}{\mathrm {d}}T$$

where subscript ${\mathrm {bp}}$ defines the boiling point (default is 373.15K), $c_{\mathrm {w}}$ and $c_{\mathrm {g}}$ are the specific heat of water and water vapour respectively. For simulation running at or near atmospheric pressure it can be assumed that the latent heat $h_{{\mathrm {fg}}}(T_p)$ is equal to the latent heat $h_{{\mathrm {fg}}}(T_{p,{\mathrm {bp}}})$ at boiling temperature 2256.4 kJ/kg.

### Devolatilization Process

During devolatilization stage the volatile matters which includes tar, light gases and pyrolysis water are released from the coal particle into the continuous phase. This process takes place if the particle temperature exceed certain defined devolatilization temperature. The devolatilization process can be modelled using expressions of different complexity. The simplest of them is the Constant Rate Devolatilization Model (CRDM) proposed by Baum and Street [102]. This model uses simple linear relationship for describing the particle mass variation during devolatilization, which can by written as

51 $$\frac{{\mathrm {d}}m_p}{{\mathrm {d}}t}=-A_0Y_{{\mathrm {vol}},0}\left( 1-Y_{{\mathrm {w}},0} \right) m_{p,0}$$

where $Y_{{\mathrm {vol}},0}$ and $Y_{{\mathrm {w}},0}$ stand for initial mass fraction of the devolatilization species and water respectively, $m_{s,0}$ is the initial mass of the coal particle and $A_0$ is the model constant which defines rate of devolatilization process. More complex is the model proposed by Badzioch and Hawksley [103] which assumes the proportionality between consumed mass and volatile matters under definition that the devolatilization rate depends only on temperature in accordance to Arrhenius law. The changing of the volatiles mass in time is described as

52 $$\frac{{\mathrm {d}}m_p}{{\mathrm {d}}t}=-k \underbrace{\left[ (1-Y_{{\mathrm {char}},0}-Y_{{\mathrm {w}},0}- Y_{{\mathrm {ash}},0})m_{p,0} \right] }_{{\mathrm {m_{\mathrm {vol}},0}}}$$

where *Y* represents the mass fraction of char, water and ash, the subscript 0 denotes initial conditions, $m_{{\mathrm {vol}},0}$ is the initial mass of volatiles fraction and *k* is the kinetic rate defined as

53 $$k=A_1{\mathrm {exp}}\left( -\frac{E}{RT_p} \right)$$

where $A_1$ represents the pre-exponential constant and *E* is the activation energy. In order to take into account the influence of rich-$\hbox {CO}_2$ atmosphere on devolatilization rate, the model of Badzioch and Hawksley [103] was extended by Yamamoto et. al [104] dividing of the volatile matters into the elements. The modified equation of the particle mass changing (52) is formulated as

54 $$\frac{{\mathrm {d}}m_p}{{\mathrm {d}}t}=-kF\underbrace{\left[ (1-Y_{{\mathrm {char}},0}-Y_{{\mathrm {water}},0}- Y_{{\mathrm {ash}},0})m_{p,0} \right] }_{{\mathrm {m}}_{{\mathrm {vol}},0}}$$

where *F* is the modification frequency factor, expressed as a function of devolatilized fraction $G_{{\mathrm {vol}}}=(m_{{\mathrm {vol}},0}-m_{{\mathrm {vol}}})/m_{{\mathrm {vol}},0}$:

55 $$F={\mathrm {exp}}\left( Y^{\mathrm {C}}_{\mathrm {vol}}G_{\mathrm {vol}} + Y^{\mathrm {H}}_{\mathrm {vol}}G_{\mathrm {vol}} + Y^{\mathrm {O}}_{\mathrm {vol}}G_{\mathrm {vol}}+Y^{\mathrm {N}}_{\mathrm {vol}}G_{\mathrm {vol}} + Y^{\mathrm {S}}_{\mathrm {vol}}G_{\mathrm {vol}}\right)$$

Other model which can be applied for devolatilization process is the Two Competing Reaction Rate Model (TCRRM) described in work of Kobayshi et al. [105]. Other models can be found in Refs. [104] and [106].

It should be pointed out that the devolatilization process in comparison to the char combustion rate is very fast. Additionally, switching regime from air- to oxy-fuel combustion should not influence the rate of devolatilization process [101]. The heat of devolatilization mainly depends on the temperature, so changing of the composition of the oxidized and combustion atmosphere should have relatively low impact on the rate of devolatilization.

### Char Combustion Process

The time and energy effect of char combustion process dominates during coal-particle burnout process. The char burning rate greatly influences the total efficiency of the power plant. In the literature a lot of the char combustion model can be found. References [107–109] are just few representative examples. However, more sophisticated models described in these contributions, are seldom used in practice. The reason for this is the high computation time and difficulties in obtaining relevant data.

### Single Reaction Model

The simplest model uses one step char-$\hbox {O}_2$ reaction where char is oxidising into the $\hbox {CO}_2$ using following reaction

56 $${\mathrm { C}}{\mathrm {(s)}}+{\mathrm {O}_{2}}\rightarrow {\mathrm {CO}_{2}}$$

The rate of the heterogeneous surface reaction are frequently described by diffusion [102] or kinetic/diffusion [101, 102] models. The first model take into account only the diffusion of oxidant to the particle surface. During carbon consumption the particle surface remain constant and the mass decrement is defined as

57 $$\frac{{\mathrm {d}}m_p}{{\mathrm {d}}t}=-4 \pi d_p D_{{\mathrm {O}_{2}},m} \frac{Y_{{\mathrm {O}_{2}}}T_f\rho _f}{S_b\left( T_p+{T}_f\right) }$$

where $D_{{\mathrm {O}_{2}},m}$ is the diffusion coefficient of oxidant in gaseous phase, $Y_{{\mathrm {O}_{2}}}$ is the mass fraction of oxygen in vicinity to particle surface and $S_b$ stands for the stoichiometric burnout ratio $(2.66\,\hbox {kg}_{{\mathrm {O}}_2}/\hbox {kg}_{\mathrm {C}})$ of the char-$\hbox {O}_2$ reaction 56. The kinetic/diffusion rate model controls the rate of char consumption on both diffusion and chemical reaction rates. This model does not account for swelling of the particle. The diameter and particle volume during carbon combustion remains constant, moreover the porous particle structure is not taken into account [107]. The particle mass variation during char combustion is formulated as

58 $$\frac{{\mathrm {d}}m_p}{{\mathrm {d}}t}=\pi d^2_p\left[ \frac{\rho _p R {T}_f Y_{{\mathrm {O}_{2}}}}{M_{{\mathrm {O}_{2}}}}\right] \frac{D_0{\mathcal {R}}}{D_0+{\mathcal {R}}}$$

where $d_p$ is the particle diameter, $M_{{\mathrm {O}_{2}}}$ and $Y_{{\mathrm {O}_{2}}}$ stand for the molecular mass and mass fraction of oxygen in vicinity to the particle surface respectively, ${\mathcal {R}}$ denotes the kinetic rate by Eq. 60 and $D_0$ is the diffusion coefficient defined in Eq. 59.

59 $$D_0= \frac{C_1}{d_p}\left[ \frac{(T_p+{T}_f)}{2}\right] ^{0.75}$$

60 $$\mathcal { R}= C_2 {\mathrm {exp}} \left( - \frac{E}{R T_p}\right)$$

where $C_1$ is the mass diffusion rate constant, and $C_2$ is the kinetic-limited pre-exponential factor. Under char combustion process the term $Q_c$ in energy balance equation of the particle (42) stands for the amount of the energy absorbed by the particle during surface combustion processes from gaseous phase. This quantity is defined as [110]

61 $$Q_c=-f_h\frac{{\mathrm {d}}m_p}{{\mathrm {d}}t}H_{{\mathrm {reac}}}$$

where $H_{{\mathrm {reac}}}$ is the heat released by the surface reaction and $f_h$ fraction of heat absorbed by the particle during surface char combustion process. When the char burnout product is $\hbox {CO}_2$ the $f_h$ is equal to 0.3.

### Heat Radiation

High temperature and presence of participating gases makes this heat exchange mode important in simulations of the energy transfer in the boiler. The gas has been modelled as an absorbing-emitting medium of spectrally dependent absorption coefficient. The scattering phenomena were due to the large dimensions of the particles in the bed, neglected. Radiation of particles has been accounted for using direct model. Spectral radiation intensity $I_{\lambda }$ at point **r** propagating in direction **s** satisfies an ordinary differential equation

62 $$\frac{{\mathrm {d}}I_{\lambda }({\mathbf {r}},{\mathbf {s}})}{{\mathrm {d}}s} = a_{\lambda }({\mathbf {r}})\left[ I_{b,\lambda }({\mathbf {r}})- I_{\lambda }({\mathbf {r}}, {\mathbf {s}})\right]$$

where $a_\lambda$ is the spectral absorption coefficient, $I_{b,\lambda }$ stands for the blackbody spectral intensity and ${\mathrm {d}}s$ is the infinitesimal length of the radiation path and $\lambda$ is the wavelength. To obtain the radiative heat source entering the energy equation, the radiation intensity should be integrated over the entire spectrum and over full solid angle. Appropriate equation reads

63 $$S_{{\mathrm {rad}}}({\mathbf {r}})=\int _{4\pi }\int _0^{\infty }a_{\lambda }({\mathbf {r}})\left[ I_{\lambda }({\mathbf {r}},{\mathbf {s}})-I_{b,\lambda }({\mathbf {r}})\right] {\mathrm {d}}\lambda {\mathrm {d}}\varOmega ({\mathbf {s}})$$

where $\varOmega$ is the solid angle. The radiative flux on the gray boundaries is evaluated from

64 $$q_{{\mathrm {rad}}}({\mathbf {r}})=\epsilon ({\mathbf {r}})\int _{2\pi }\int _0^{\infty }\left[ I_{\lambda }({\mathbf {r}},{\mathbf {s}})-I_{b,\lambda }({\mathbf {r}})\right] \cos ({\mathbf {r}},{\mathbf {s}}){\mathrm {d}}\lambda {\mathrm {d}}\varOmega ({\mathbf {s}})$$

where $\epsilon$ stands for emissivity and $\cos ({\mathbf {r}},{\mathbf {s}})$ stands for the cosine of the angle made by surface normal and the direction of the incident radiation. The integration over angle is performed using the discrete ordinate (DO) method [111]. The spectral radiation properties of the optically active gases i.e., $\hbox {CO}_2$ and $\hbox {H}_2\hbox {O}$ have been performed using the Weighted Sum of Gray Gases (WSGG). In this approach, the radiation of gases is approximated by a finite number of gray gases of different absorption coefficients and one transparent gas. The most accurate at theoretically sound procedure of evaluating the values of the WSGG coefficient is to use the spectral emissivity data of $\hbox {CO}_2$ and $\hbox {H}_2\hbox {O}$ molecules stored in appropriate database like HITEMP 2010 [112]. The procedure of computing the radiation constants is cumbersome and time consuming. The data available in Ansys FLUENT are proposed by Smith [113]. These data are limited to the ratio of $p_{\mathrm {H_2O}}/p_{\mathrm {CO}_{2}}$ equal to 1 and 2. Several research groups are focused on examining the impact of oxy-fuel atmosphere on radiative transfer and developing computation tools suitable to deal with the ratio of $p_{\mathrm {H_2O}}/p_{\mathrm {CO}_{2}}$ smaller than one, as it is the case in oxy-combustion [114–116].

The developed OXY WSGG model takes into account changes of the molar fraction ratios of $\hbox {H}_2\hbox {O}$ to $\hbox {CO}_2$ $(M_{{\mathrm {rad}}}=p_{{\mathrm {H_2O}}}/p_{{\mathrm {CO}}_2})$ along the combustion chamber in range from 0.01 to 4.0 for a temperature range of 300–2400 K. The standard WSGG model [113] assumes constant value of the molar fraction ratio $M_{{\mathrm {rad}}}$ which is seen as source of error in the oxy-fuel combustion modelling. To overcome this problem the OXY WSGG model has been implemented to the calculation procedure using set of the user defined functions for calculating emissivity weighting factor and absorbtion coefficient. The total emissivity in the OXY WSGG model is defined as [117]

65 $$\varepsilon _t=\sum ^{N_g}_{k=0}a_k\left( 1-e^{-K_kp_ts}\right)$$

where $a_k$ is the emissivity weighting factors, $K_k$ absorption coefficients, $N_g$ stands for the number of gray gases in the model, $p_t$ is the sum of the partial pressure $p_t=p_{\mathrm {H_2O}}+p_{\mathrm {CO}_{2}}$, and *s* stands for the path length. In OXY WSGG model four gray gases was used $(N_g=4)$ [116, 118] with a transparent gas $k=0$. In the WSGG model the sum of the weighted factors has to be equal to unity as

66 $$\sum ^{N_g}_{k=0}a_k=1$$

whereas the weighting factor for the transparent gas is defied as

67 $$a_0=1-\sum ^{N_g}_{k=1}a_k$$

In standard WSGG model [113] implemented in Ansys FLUENT [1] the weighting factors are defined as the function of temperature only, moreover the absorption coefficients are constant. The developed OXY WSGG model [119] contrary to standard WSGG model defines the weighting factors Eq. 68 as a polynomial function of the temperature and molar fraction ratio $(M_{{\mathrm {rad}}})$, additionally in this model the absorption coefficients in Eq. 65 are also considered as a polynomial function of the molar fraction ratio, see Eq. 70.

68 $$a_k=\sum ^{4}_{j=0}b_{k,j}T^j_r$$

where $T_r$ is the normalized temperature defined as the ratio of *T* to $T_{{\mathrm {ref}}}$, the polynomial function $b_{k,j}$ is calculated as

69 $$b_{k,j}=\sum ^{4}_{i=0}C_{k,j,i}M^i_{{\mathrm {rad}}}$$

where $C_{k,j,i}$ are the model coefficients. The absorption coefficient of gray gases in Eq. 65 is defined as

70 $$K_k=\sum ^{4}_{i=0}d_{k,i}M^{i}_{{\mathrm {rad}}}$$

where $d_{k,i}$ are the model coefficients. The evaluated model coefficients $C_{k,j,i}$ and $d_{k,i}$ based on emissivity database HITEMP 2010 for $\hbox {CO}_2$ and $\hbox {H}_2\hbox {O}$ are shown in Table 1, [119]. The proposed OXY WSGG model was also used for modelling oxy-fuel combustion process in retrofitted large scale CFB boiler (460 MWe). While for air combustion standard WSSG model implemented in Ansys FLUENT has been used, the OXY WSSG model has been implemented by a set of UDFs.

**Table 1:** OXY WSGG model coefficients

Coeff.	k	j	i = 0	i = 1	i = 2	i = 3	i = 4
C	1	1	0.7412956	−0.5244441	0.582286	−0.2096994	0.0242031
C	1	2	−0.9412652	0.2799577	−0.7672319	0.3204027	−0.0391017
C	1	3	0.8531866	0.0823075	0.528943	−0.2468463	0.031094
C	1	4	−0.3342806	0.1474987	−0.4160689	0.1697627	−0.0204066
C	1	5	0.0431436	−0.0688622	0.1109773	−0.0420861	0.0049188
D	1	–	0.0340429	0.0652305	−0.0463685	0.0138684	−0.001445
C	2	1	0.1552073	−0.4862117	0.3668088	−0.1055508	0.0105857
C	2	2	0.6755648	1.409271	−1.383449	0.457521	−0.0501976
C	2	3	−1.125394	−0.5913199	0.9085441	−0.3334201	0.0384236
C	2	4	0.6040543	−0.0553385	−0.1733014	0.0791608	−0.0098934
C	2	5	−0.1105453	0.0464663	−0.0016129	−0.0035398	0.0006121
D	2	–	0.3509457	0.7465138	−0.529309	0.1594423	−0.0166326
C	3	1	−0.0345199	0.2656726	−0.1225365	0.0300151	−0.0028205
C	3	2	0.4112046	−0.572835	0.292449	−0.0798076	0.0079966
C	3	3	−0.5055995	0.4579559	−0.2616436	0.0764841	−0.0079084
C	3	4	0.2317509	−0.1656759	0.1052608	−0.0321935	0.003387
C	3	5	−0.0375491	0.022952	−0.0160047	0.0050463	−0.0005364
D	3	–	109.8169	−50.92359	23.43236	−5.163892	0.4393889
C	4	1	0.2550242	0.3805403	−0.4249709	0.1429446	−0.0157408
C	4	2	−0.6065428	0.3494024	0.1853509	−0.1013694	0.0130244
C	4	3	0.8123855	−1.102009	0.4046178	−0.0811822	0.00062981
C	4	4	−0.453229	0.6784475	−0.3432603	0.0883088	−0.0084152
C	4	5	0.0869309	−0.1306996	0.0741446	−0.0202929	0.002011
D	4	-	4.570740	2.168067	−1.498901	0.4917165	−0.0542999

#### Particulate Effect in the Radiation Model

As it has been mentioned earlier due to large dimensions of the particles in the bed, the gas scattering coefficient in Radiative Transfer Equation (RTE) is neglected. In order to take into account the influence of particulate phase on radiative heat transfer the RTE in Ansys FLUENT is defined as [1]

71 $$\nabla \cdot \left( I{\mathbf {s}}\right) + \left( a+a_p+\sigma _p\right) I\left( {\mathbf {r}},{\mathbf {s}}\right) = a n^2\frac{\sigma T^4}{\pi } + E_p + \frac{\sigma _p}{4\pi }\int ^{4\pi }_{0} I\left( {\mathbf {r}},{\mathbf {s}}\right) \phi \left( {\mathbf {s}},{\mathbf {s}}'\right) d\varOmega '$$

where *a* is the absorption coefficient, *n* refractive index, ${\mathbf {r}}$ position vector, ${\mathbf {s}}$ direction vector, ${\mathbf {s}}'$ scattering direction vector, $\phi$ is the phase function, $\varOmega '$ is the solid angle. The $a_p$ is the equivalent absorption coefficient, $E_p$ is the equivalent emission, and $\sigma _p$ is the equivalent particle scattering factor due to the presence of particles. Those equivalent factors are defined as

72 $$a_p= \sum ^N_{n=1}\varepsilon _{pn}\frac{A_{pn}}{V}$$

73 $$E_p= \sum ^N_{n=1}\varepsilon _{pn}A_{pn}\frac{\sigma T^4_{pn}}{\pi V}$$

74 $$\sigma _p= \sum ^N_{n=1}(1-\varepsilon _{pn})(1-f_{pn})\frac{A_{pn}}{V}$$

where $T_{pn}$, $A_{pn}$, $f_{pn}$, and $\varepsilon _{pn}$ are the temperature, projected surface, scattering factor, and emissivity of the particle *n*, *N* is the total number of particles in given volume *V*.

## Experimental Facility of the Circulating Fluidized Bed

**Fig. 10:**
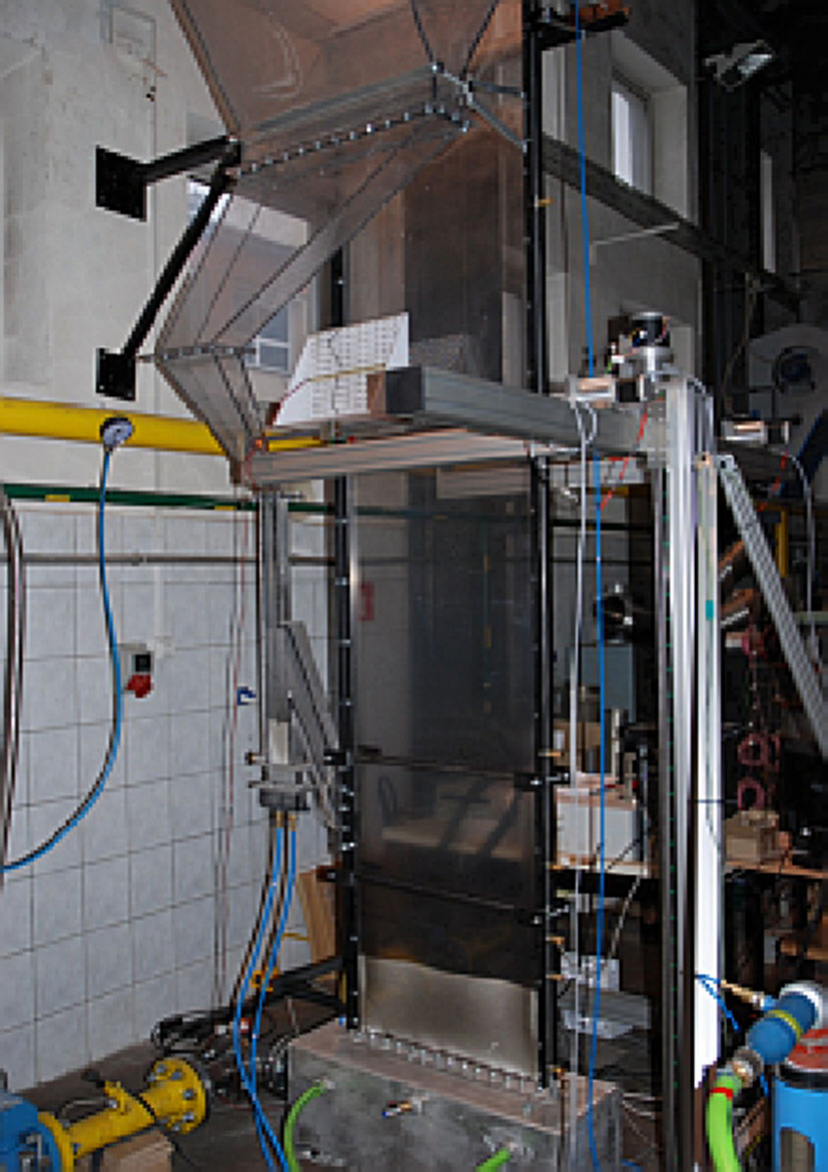
Front view on experimental facility (*left*) and fluidization process (*right*)

An experimental rig aimed at validation of Euler–Euler and DDPM techniques and some submodels has been designed and built at the Institute of Thermal Technology (ITT). The idea behind building the rig was to validate the numerical procedures and test their robustness in a relatively simple environment without introducing sophisticated combustion models and 3D flow i.e., following the philosophy not to introduce too many complexities at one step. When designing this facility, the experience gained at Abo Academy in Turku, Finland when running a similar rig, has been utilized [7, 8]. Figure 10 shows the 3 m high, 0.6 m wide ITT installation.

The side walls of the solid separator and the walls of the standpipe have been made of polycarbonate plates, whereas the bed frame has been made of aluminium profiles. The fluidization gas from aeration box has been distributed by 13 equally spaced gas nozzles. The installation has been wider than its Abo prototype, the recirculation section, particle separator and aeration box have been completely redesigned. Small distance between walls of the standpipe (0.017 cm) makes the 2D approximation of the flow realistic. The transparent walls and small depth allows for visual observation of the flow.

### Experimental Facility

**Fig. 11:**
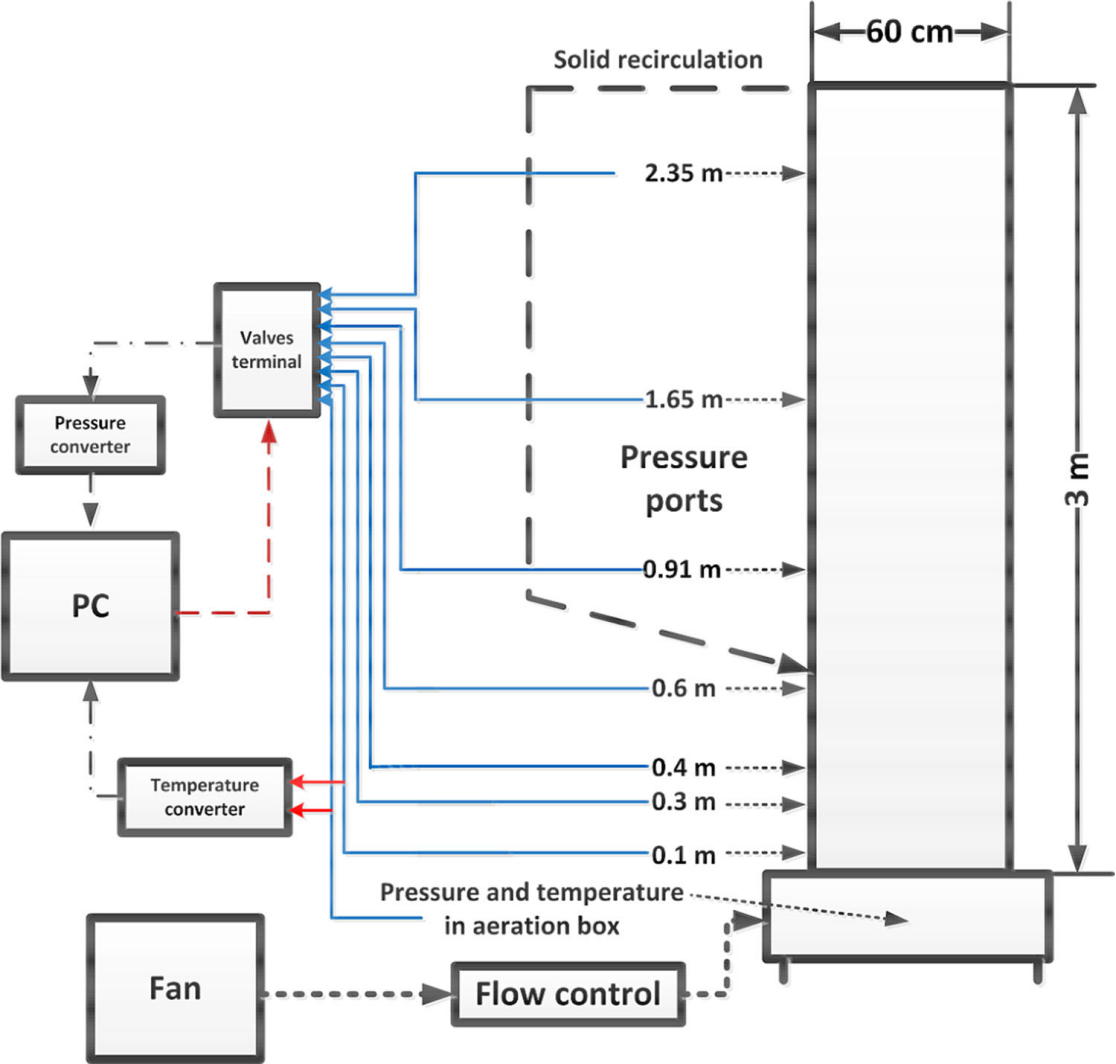
Scheme of the circulating fluidized bed experimental facility

The scheme of the experimental facility is shown in Fig. 11 where the positions of the pressure ports and used equipment’s are marked. The static pressure has been measured at 7 ports along standpipe using the pressure transducer (*Fuji Electric FCX-AII V5*) connected with valve terminal (8 solenoid valves *Danfoss 18 W*), and a PC via a pressure converter (direct current (DC)-pressure) (*Czaki LM-220*). The communication between the pressure converter and the PC has been through the RS-232 communication ports. During the experiment the pressure has been measured periodically at specified time intervals. The response time of the solenoid valve has been controlled by a microprocessor via the RS-232 communication ports, and set of transmitters connected with valves and power supply. In order to calculate the velocity of the gas above the distributor, the temperature within the aeration box, and 0.1 m above the gas distributor have been measured using thermocouples connected to temperature transducer, *Czaki EMT-140*. The flow rate of the fluidization gas delivered by the fan *Venture Industries DSC40A1100T* to the aeration box has been measured by a turbine flow-meter *Common* equipped with temperature and pressure sensors. For monitoring, controlling and acquisition of experimental data an *in-house* software has been written in LabVIEW 2012 environment [120].

### Experiment

Spherical glass particles have been used as fluidization material. The overall mass of the glass particles has been equal to 5 kg. Table 2 presents the particle size distribution along with its Satuer diameter. The density of the particles $(2478\,\hbox {kg/m}^3)$ has been measured using *in-house* porosity measuring device [121]. The experiments have been carried out for the four gas flow rates listed in Table 3. For all investigated cases highly turbulent bed has been observed. The amount of solid material in the recirculation section has been weighted at an instance when the gas flow in both, recirculation section and gas box were simultaneously and abruptly cut off. The ambient pressure has been equal to 975.3 hPa. The gas pressure shown in Table 3 has been calculated for measured static pressure in port located 0.1 m above the wind box. Severe difficulties have been experienced by static electricity generated as a result of interaction of polycarbonate walls and glass particles. To overcome this difficulties the glass particles before they have been inserted into the facility, were mixed with antistatic additives *Larostat*, and *Neostatic*.

**Table 2:** The PSD used as a fluidization material

Particle range (μm)	Mean size (μm)	Particle mass (kg)	Mass fraction (Y_d_)
100–200	150	1.000	0.20
200–300	250	1.101	0.22
300–400	350	2.167	0.43
500–630	565	0.730	0.14
Sauter diameter	269 μm

**Table 3:** Measured normal gas flow rates and calculated parameters

	Mass flow rate (kg/s)	Ambient pressure (Pa)	Gas temperature (°C)	Gas (kg/m^3^)	Velocity, density (m/s)	Mass (kg)
Case A	1.97·10^−2^	100359	17.4	1.2072	1.605	4.8
Case B	2.84·10^−2^	100359	20.4	1.1915	2.341	4.5
Case C	3.18·10^−2^	100046	23.1	1.1772	2.650	4.3
Case D	3.79·10^−2^	99566	25.6	1.1614	3.202	3.8

### Numerical Model

**Fig. 12:**
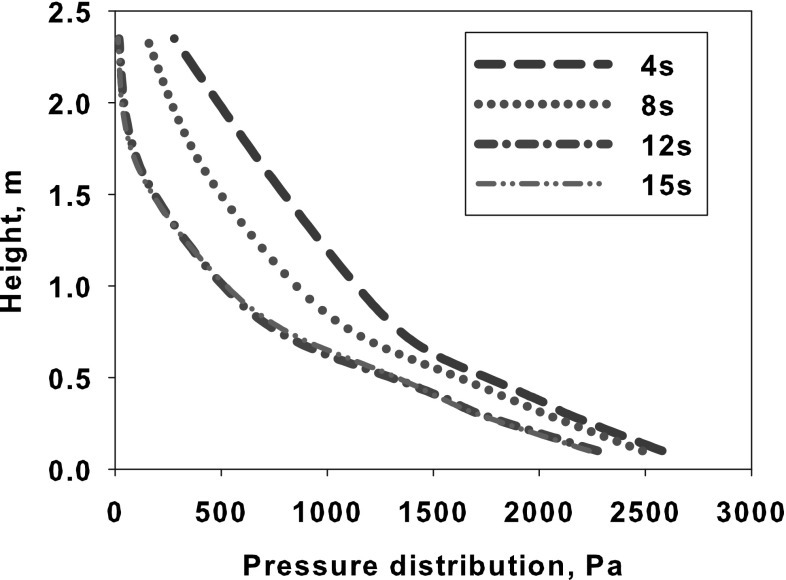
Influence of averaging time on evaluated average pressure distribution in fluidized bed

The geometry of the experimental facility has been simplified to 2D model and both the particle separator and recirculation section have been replaced by set of user defined functions written for Euler–Euler and hybrid Euler–Lagrange approaches. Transient simulations have been performed for 10 s to obtain a quasi-steady state, and then for another 10 s for collecting the time averaged data. The influence of the averaging time on predicted pressure distribution in fluidized bed is shown in Fig. 12. It can be seen that between 8 and 12 s the pressure distribution has had stabilized. The simulation time step size has been 0.001 s for both used models ensuring convergent solution in subsequent time steps. The closure models used in the transport equation are shown in Table 4. This collection of models has been selected after a number of numerical tests using simple 2D cases. For the sake of brevity, the report of these trials has not been included in the dissertation.

The numerical mesh has been built of 18,338 quad elements. Due to the long computing time where 20 s of simulation has taken approximately 40 days of wall clock time for Euler–Euler (one solid phase) and 28 days using hybrid Euler–Lagrange approach. The details concerning the mesh generation procedure for hybrid Euler–Lagrange can be found in Sect. 4.3.1. For hybrid Euler–Lagrange simulations, the PSD has been represented using the Rosin–Rammler distribution parameters. The calculated mean diameter has been equal to $289\,\upmu \hbox {m}$ with spread parameter 2.28. The maximum and minimum particle diameters have been equal to $630\,\upmu \hbox {m}$ and $100\,\upmu \hbox {m}$, respectively. Using Euler–Euler approach the PSD has been represented by Sauter mean diameter using one dispersed phase equal to $289\,\upmu \hbox {m}$.

**Table 4:** Closure models for 2D cases

Solid pressure	Lun et al. [37]
Solid viscosity	Gidaspow [30]
Bulk viscosity	Lun et al. [37]
Friction viscosity	Schaeffer [95]
Friction pressure	Based on KTGF [36]
Granular temperature	Algebraic [49]
Drag model	Gidaspow [30]
Restitution coeff. (*e*_*ss*_)	0.9
Friction limit ($\varepsilon ^{fr}_s$)	0.60
Package limit ($\varepsilon ^*_s$)	0.62
Wall restitution coeff. DDPM	$e_{{\mathrm {nor.}}}=0.9$; $e_{{\mathrm {tan.}}}=0.9$
Transition factor DDPM	1.0

#### Mesh Generation for Hybrid Euler–Lagrange Approach

**Fig. 13:**
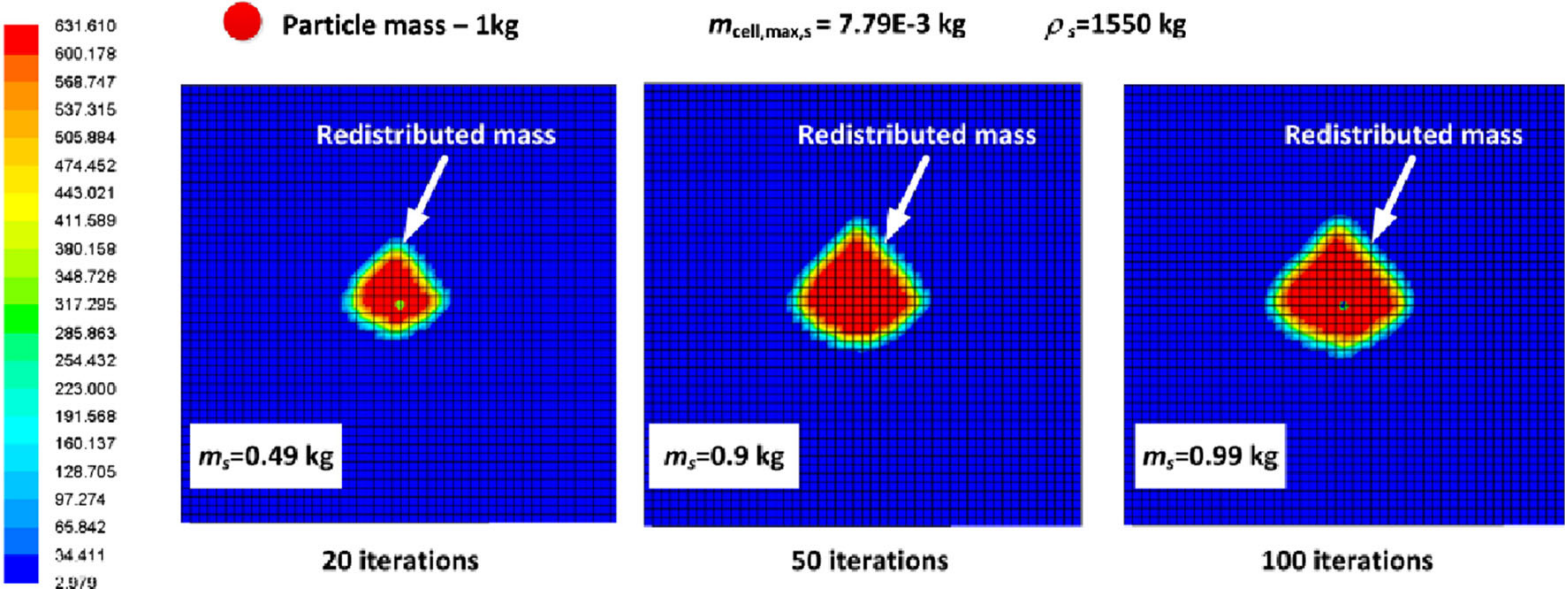
Distribution of the solid material over numerical cells

Generation of mesh for hybrid Euler–Lagrange required special attention. For obvious reasons, the amount of solid particles within a given numerical cell cannot exceed a package limit $\varepsilon ^*_{s}$ which in default model settings is equal to 0.63. The maximum cell mass capacity can be calculated from a simple relation

75 $$m_{{\mathrm {cell,max}},s} = \varepsilon ^*_{s} \rho _s V_{\mathrm {cell}}$$

where $\rho _s$ is the density of solid material and $V_{\mathrm {cell}}$ is the volume of the computational cell. In the course of Lagrangian particle tracing, the maximal mass capacity of the cell may be exceeded. In such cases, the excessive mass has to be redistributed over neighbouring cells. Figure 13 shows how a large mass (1 kg) of particles located at the center of computational domain, is iteratively redistributed over neighbouring cells. If the number of iteration within the continuous phase is too small, the masses evaluated in the Lagrangian frame of reference and in Eulerian grid can differ. It is thus important to control the number of iterations so that the solid mass in the continuous and disperse phases are enough. The number of iterations in the continues phase can be reduced by increasing cell size, as then a given cell may accommodate more particles. This however, deteriorates the accuracy of predicting other flow variables. In order to keep the mass calculated in the Lagrangian and Eulerian frame of reference close to one another, both masses have to be controlled during the solution procedure. To maintain the accuracy of mass balance special UDFs have been written. These procedures have been executed at the end of each time step. The number of iterations in the continuous phase, has been controlled by the difference of mass balances. If the difference exceeded 10% the calculations have been terminated with an appropriate error message.

**Fig. 14:**
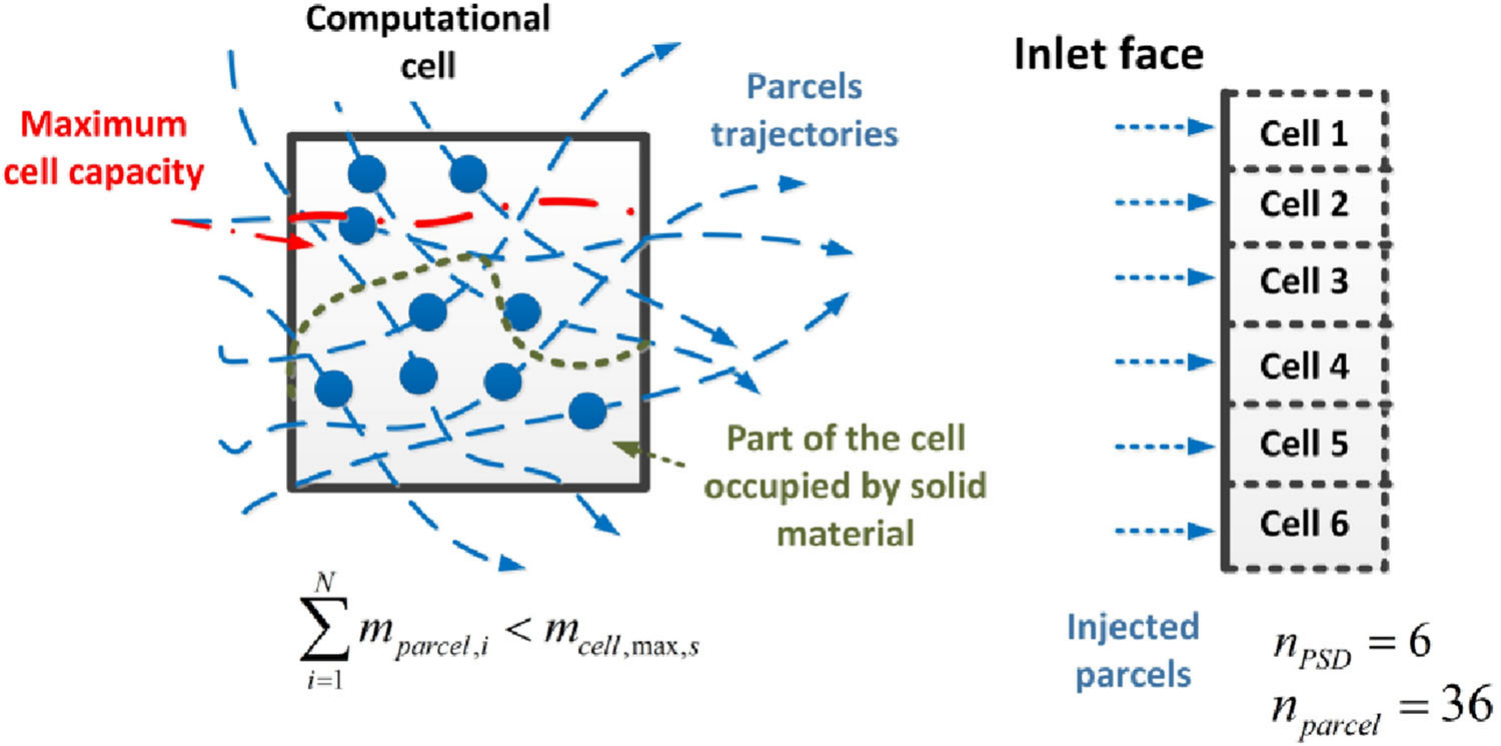
Cell capacity occupied by migrated parcels (*left*), injected amount of parcels (*right*)

As already mentioned, the maximum cell capacity may be exceeded during simulation by accumulation of a large number of particles in one computational cell. This situation is illustrated in Fig. 14 (left). Similar situation can take place when the injected mass exceeds the mass capacity of the cells adjacent to an inlet face, see Fig. 14 (right). This problem can readily be cured by uniform redistribution of the solid material over injected parcels by using UDFs. The number of the injected parcels can be calculated from

76 $$n_{\mathrm {inj,parcel}}=n_{\mathrm {PSD}}n_{\mathrm {cell}}$$

where $n_{\mathrm {cell}}$ is the number of cells adjacent to the inlet and $n_{\mathrm {PSD}}$ defines the number of characteristic diameters used for describing PSD. For a known number of the injected parcels, the mass of individual parcel can be calculated as

77 $$m_{\mathrm {parcel}}=\frac{m_{{\mathrm {inj}}}}{n_{\mathrm {inj.,parcel}}}$$

where $m_{{\mathrm {inj}}}$ is the overall mass of injected material in one time step. Figure 14 (right) illustrates the case of an injection face, where six cells belong to the inlet and the PSD has been represented by six diameters. In this case the injected mass will be redistributed into 36 parcels.

#### Recirculation Procedure for 2D Experimental Facility

**Fig. 15:**
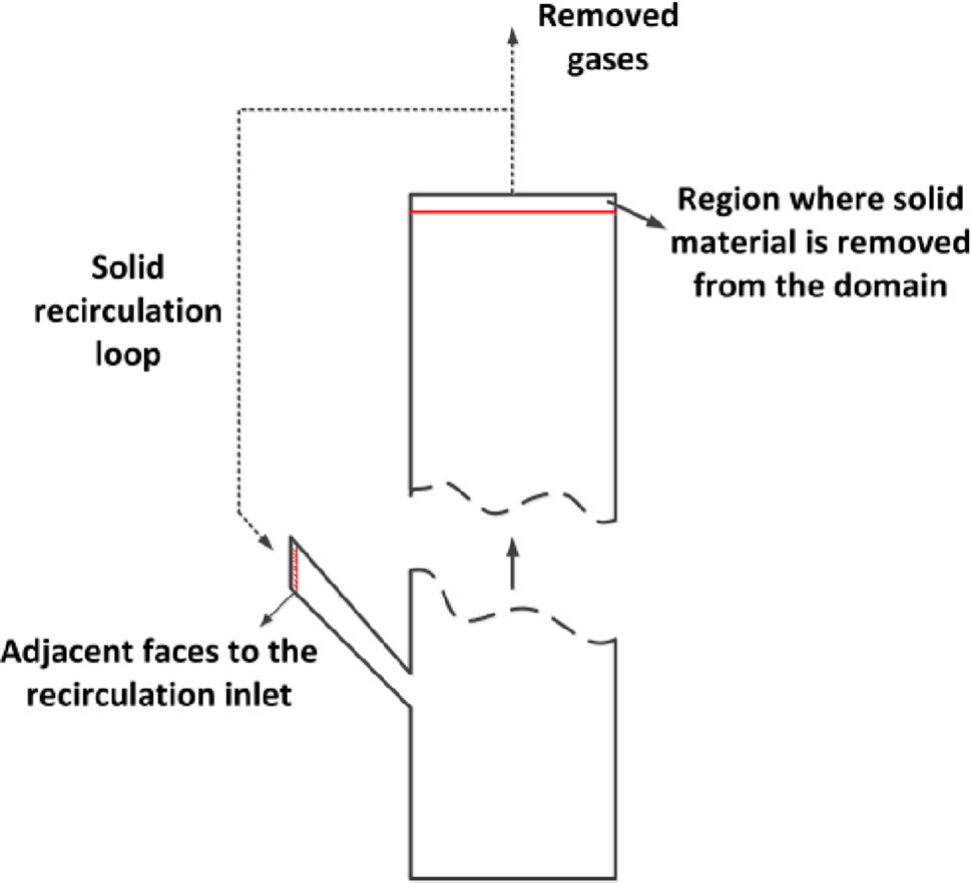
Recirculation procedure

The schematic diagram of recirculation procedure is shown in Fig. 15. The material collected at the upper part of the fluidized bed has been recirculated back to the domain via the recirculation inlet located at the left side of the standpipe. The recirculated mass has been split into a several parcels streams entering then the solid recirculation port. During simulation the overall number of tracked parcels by hybrid Euler–Lagrange approach has been kept between six and eight millions. Below this parcel numbers for generated mesh size, the intensive particle clustering has been observed in the upper part of the domain. For lower parcels number, the disagreement between calculated masses based on mass of injected parcels and those calculated using solid volume fraction has not been acceptable.

In the Euler–Euler simulations the collected mass at the outlet of the standpipe has been directly injected through the recirculation inlets. The recirculation procedure for the Eulerian approach has been very simple in comparison to the hybrid Euler–Lagrange where complex procedure has to be used for dispersed phase. The reason for this has been the monodisperse treatment of the solid phase in Euler–Euler simulations.

### Sample Results

**Fig. 16:**
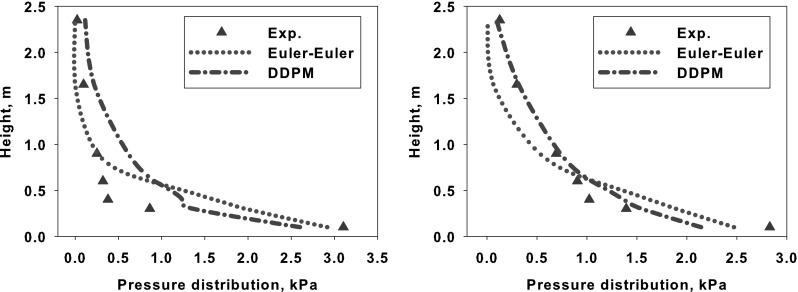
Comparison of the pressure distribution for the *case A* (*left*), and *case B* (*right*) in the CFB evaluated by the Euler–Euler and DDPM approaches against experimental data

Figures 16 (left, right) and 17 (left, right) shows the comparison of calculated pressure distribution in the cold experimental facility using Euler–Euler and hybrid Euler–Lagrange approaches against experimental data. The results have shown good agreement between both used numerical approaches. The discrepancies between numerical and experimental data can be attributed to the distribution of solid material within the facility. A significant gradient in the distribution of the solid material influences the precision of the evaluation of the granular temperature. This in turn degrades the precision of the particle interactions and thus the pressure drop.

**Fig. 17:**
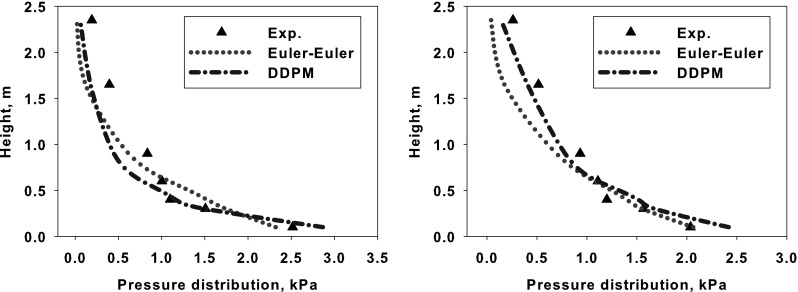
Comparison of the pressure distribution for the *case C* (*left*), and *case C* (*right*) in the CFB evaluated by the Euler–Euler and DDPM approaches against experimental data

## Large Scale CFB Boiler: 460 MWe

**Fig. 18:**
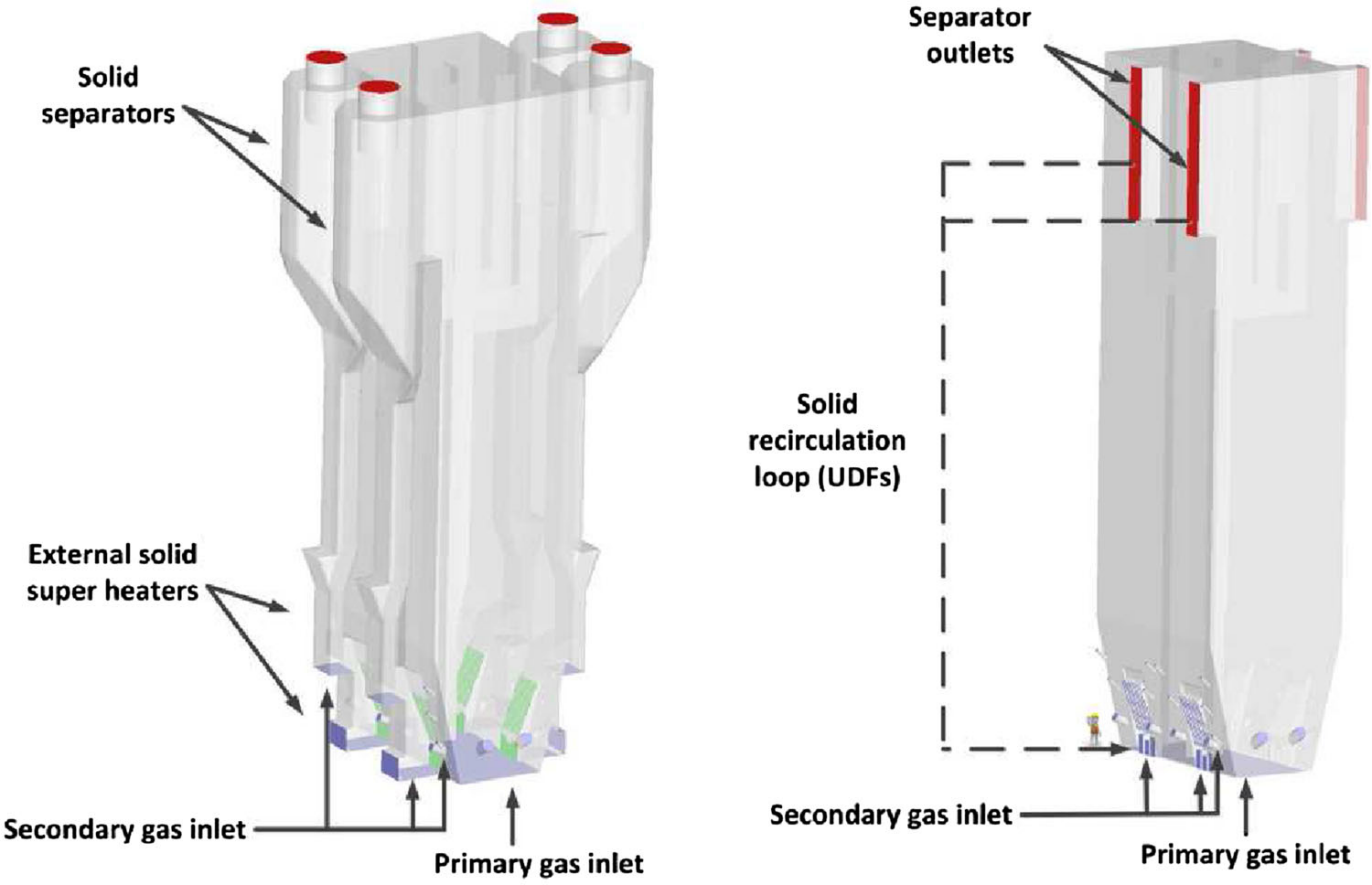
Complete geometry of the Lagisza boiler (*left*), and simplified model with marked recirculation loop

The particle transport was considered and modelled selecting the large industrial CFB boiler installed in Poland (see Fig. 18). For that purpose the commercial Ansys FLUENT 15.0 commercial CFD package, augmented with set of user defined functions (UDF) into the solution procedure. User functions were applied, namely the simulation of material recirculation (simplified boiler geometry), calculating distribution of suspension density, implementing drag model, and for many other thinks. Implemented drag model was used to ensure smooth transition between dense and dilute solid ranges in CFB boiler. At the present stage of the modelling, the computational domain of the boiler was limited to one half of the combustion chamber. Moreover, in order to reduce the calculation time, the computational domain was limited to the combustion chamber only with solid and gas injection ports, see Fig. 18.

### Geometry and Mesh of the CFB Boiler

The geometry of the investigated CFB boiler, operating in a power plant in Poland consisted of 8 solid separators (four at each boiler side walls) which have been connected with the boiler combustion chamber using External Solid Super Heaters (ESSH). The rectangular cross section of the combustion chamber is 27.6 × 10.6 m while the combustion chamber height is 48 m. The boiler geometry is equipped with 16 secondary gas inlets and 7 fuel feeding ports distributed at both boiler side walls. At the left wall two solid injection ports were located. To simplify the geometry of the primary inlet it was assumed that this air enters the chamber through its whole bottom cross section. The boiler geometry was meshed using total number of cells equal to 1,300,000. The mesh sensitivity study was described in [26].

**Fig. 19:**
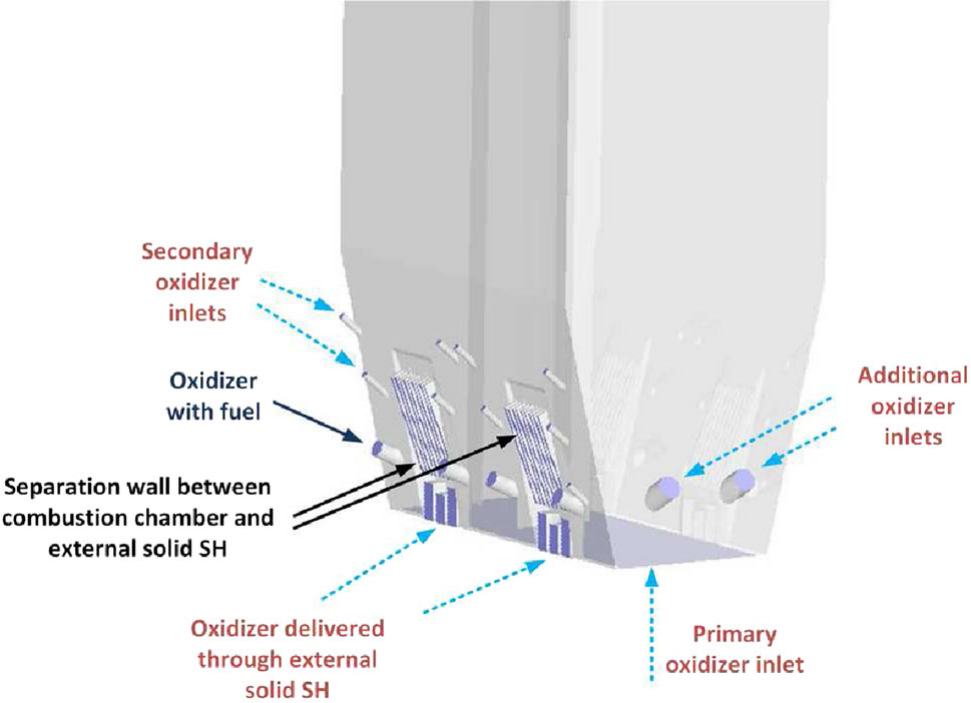
Marked inlets at the bottom part of the Lagisza boiler

To not introduce additional complexities with the geometrical model, the water tubes on the boiler walls were simplified to flat steel walls with assigned internal wall temperature equal to 560 °C (temperature of the evaporated water), thickness, material properties and prescribed emissivity on the side of combustion chamber. The internal emissivity of the wall was set to 0.95 and no-slip condition was used. The specified oxidizer inlets are depicted in Fig. 19 where also the thickness of the water tubes were shown. The oxidizer temperature for the primary and secondary inlets and those entering through the fuel injection ports was equal to 230 °C. The temperature of oxidizer delivered through the external solid super-heaters (SH) inlets was 800 °C. The composition of as received (AR) coal is collected in Table 5. The material properties of coal, sand and ash are presented in Table 6.

**Table 5:** Ultimate and proximate analysis of the burned coal (AR basis)

Proximate (%)		Ultimate (%)	
Ash	19.755	C	53.653
Water	14.32	H	3.601
Vol	30.7	S	1.370
Char	35.23	N	1.1
		O	6.201
Low heating value (LHV_AR_)		20 113 kJ/kg

**Table 6:** Material properties for fluidization material for air- and oxy-fuel combustion

	Coal	Ash	Sand
Density (kg/m^3^)	1300	2000	2000
Mean diameter (μm)	322	325	222
Max diameter (μm)	2000	2000	600
Min diameter (μm)	50	50	50
Spread parameter	0.7	0.7	2.35
Number of diameters	6	15	15

During simulations the ash material was recirculated using UDFs through the external solid SH inlets, whereas the sand was injected through additional oxidizer inlets located on the wall of the combustion chamber, see Fig. 19. Sand was used to initialize the fluidization process and to compensate the material losses in order to keep the amount of the solid material constant within the boiler. The amount of solid material within the combustion chamber was controlled by UDFs. Table 7 shows the amount of the injected fuel and oxidizer for air- and oxy-fuel combustion. The amount of burned coal was calculated based on known boiler thermal load (864 MW) and coal heating value shown in Table 5. The overall amount of oxidizer delivered to the air- and oxy-fuel combustion process was calculated using the fuel composition and value of excess oxygen ratio equal to 1.35. The simulation of oxy-fuel combustion process should be seen as a theoretical model. This simulation was performed only to check the response of the existing CFB boiler to the change of the operating regime from air- to oxy-fuel combustion. It was assumed that the mole fraction of the oxygen in the oxidizer after mixing the technical oxygen $\hbox {O}_2/\hbox {N}_2$ (95/5) with flue gases should be equal to 30 %. Such amount of oxygen in oxidizer mixture produces similar amount of oxidizer delivered to oxy-fuel combustion process as in the case of conventional air firing. Additionally, for oxy-fuel combustion it was assumed that the recycled flue gases were dried before mixing with the technical oxygen. The compositions of the oxidizer mixture for both investigated case are shown in Table 8. The coal combustion process was modelled using two step reaction mechanism where within first step the CO is generated, whereas the second step convert the produced CO to $\hbox {CO}_2$. The char combustion was controlled using one step heterogeneous reaction.

**Table 7:** Amount of injected coal and oxidizer for combustion process in large scale CFB unit

	AIR	OXY
Coal mass flow rate (kg/s)	21.5	21.5
Oxidizer mass flow rate (kg/s)	209.4	199.5

**Table 8:** Oxidizer composition for air- and oxy-fuel combustion

	AIR (kg_i_/kg_oxi_)	OXY (kg_i_/kg_oxi_)
$\hbox {O}_2$	0.2308	0.2417
$\hbox {CO}_2$	0.0	0.7156
$\hbox {H}_2$O	0.0012	0.0
$\hbox {N}_2$	0.7680	0.0426

To take into account the influence of turbulence onto the combustion process the Eddy Dissipation Model (EDM) [122]. The Discrete Ordinate (DO) radiation model was chosen for modelling radiation [111]. The gray gas model defined as WSGG proposed by Smith [113] was used to determine absorption coefficient for air firing process, whereas for modelling oxy-fuel combustion the model parameters for the WSGG were calculated using the *OXY WSGG* model. Due to lack of the kinetic data for the burned coal the constant rate devolatilization and kinetic/diffusion surface char combustion model was used. The model constants, namely the mass diffusion-limited rate constant and kinetic-limited rate (pre-exponential factor) were adjusted iteratively during the simulations. They were selected under assumption that the injected coal particles should burn before they will live computational domain. The final model constants were equal to 4$\times 10^{-12}$ and 0.002, respectively. In the future simulations the exact kinetic data should be used for both, air- and oxy-fuel combustion process. At present, for the burned coal such data was not available.

### Sample Results

**Fig. 20:**
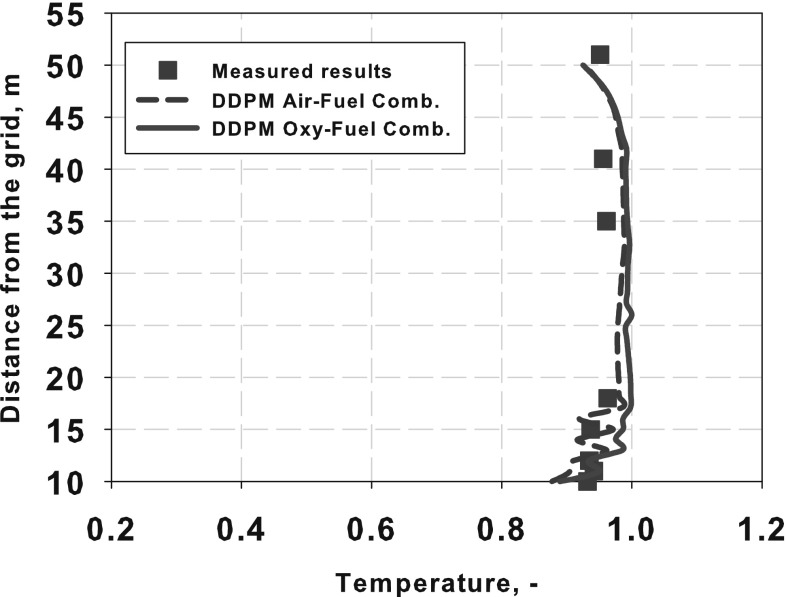
Time averaged scaled temperature profile compared with measured data for air- and oxy-fuel combustion

**Fig. 21:**
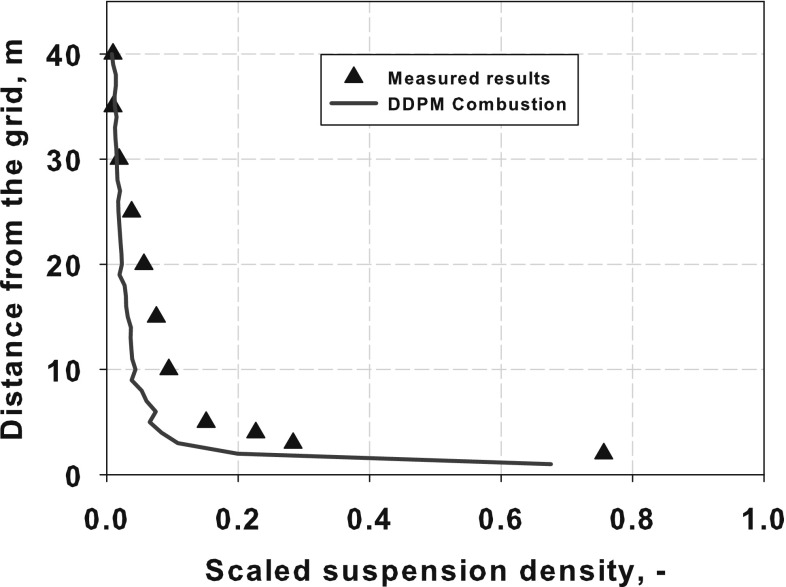
Time average distribution of material in the combustion chamber carried out for *pseudo* combustion and air-fuel combustion

**Fig. 22:**
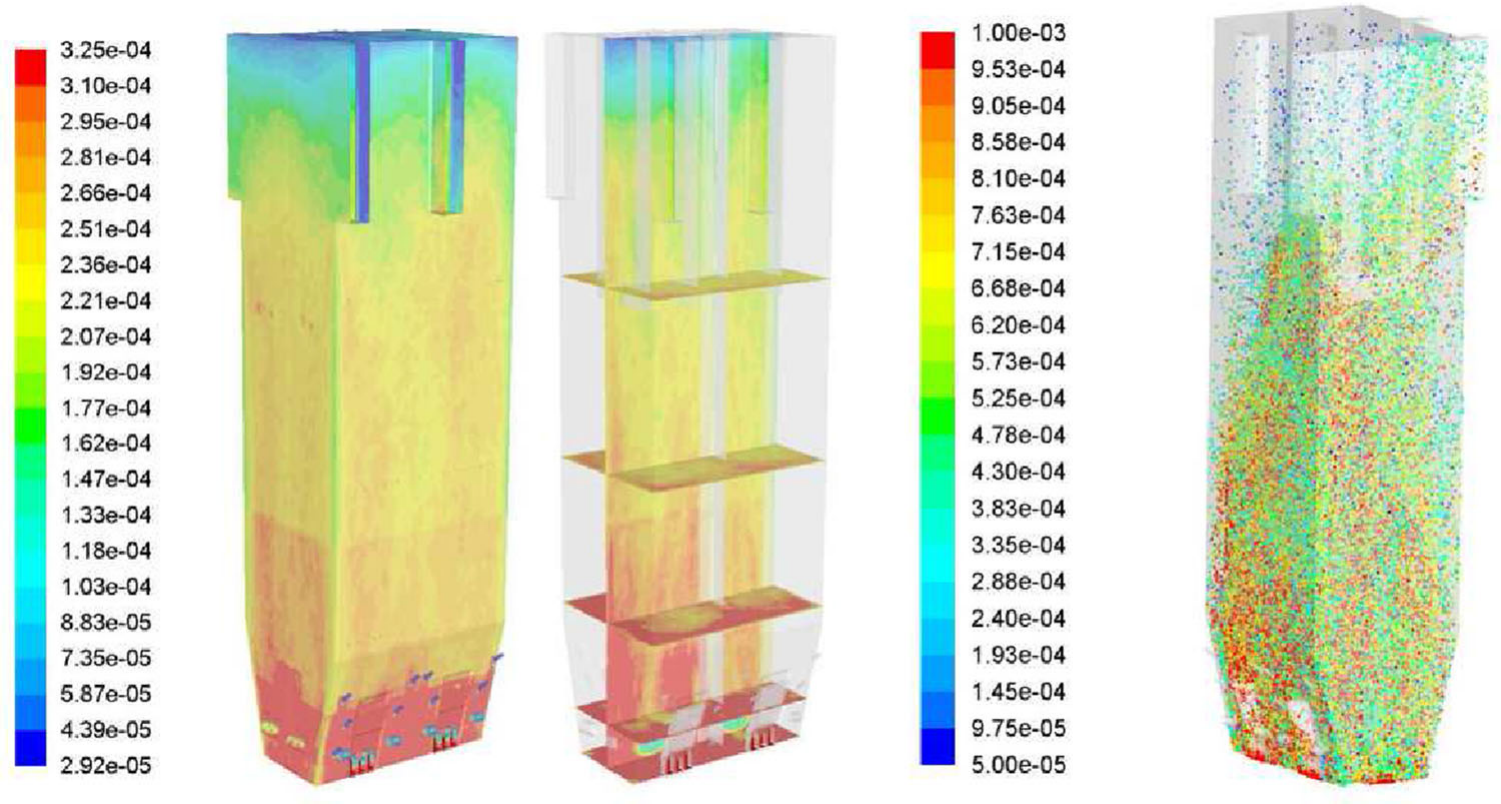
Calculated time averaged distribution of particle size (m) on the external boiler walls (*left*) and within the boiler (*right*)

Figure 20 shows the comparison of the calculated and measured scaled temperature profiles for air- and oxy-fuel combustion processes. In case of radiation modelling, the absorption coefficient for gas emissivity was calculated using advance model for calculating properties of radiative active gases [123, 124] which was earlier used for modelling combustion process under oxy-fuel combustion within lab-scale experimental test rig [25]. It can be seen that the time averaged temperature profile for oxy-fuel combustion case was higher than this calculated for conventional air-firing process and measured data. Moreover, at the upper part of the boiler, the temperature profile for both investigated cases bended from straight perpendicular line. This situation was caused by the high velocity of flue gases due to the narrow outlet and high mass flow rate of gases to separators. This influenced on the hydrodynamic behaviour and heat transfer process at the upper section of the boiler was observed. In order to resolve this problem, external solid separator and the recirculation loop should be included in the model. Nevertheless, taking into account complete geopolitical model with some simplification of the model cause that the simulation time considerably increase [27]. Figure 21 illustrates the dimensionless distribution of the solid material along the boiler high. The calculated particle size distribution within the boiler is illustrated in Fig. 22 (left), whereas some particle distribution over combustion chamber for selected simulation time is depicted in Fig. 22 (right). The results show that the particles larger than $300\,\mu \hbox {m}$ have been mainly collected at the bottom of the combustion chamber, which is in line with the measured particle size distribution.

## Large Scale CFB Boiler: 260 MWe

**Fig. 23:**
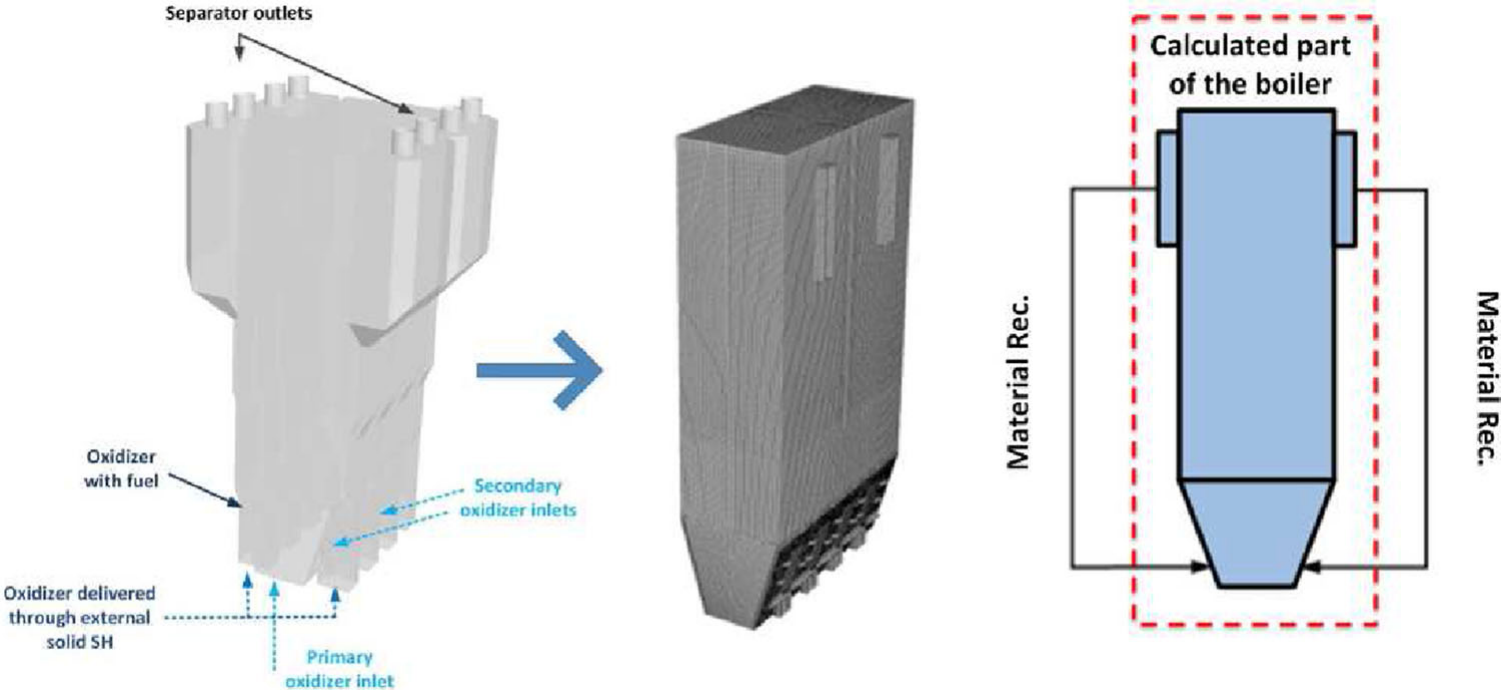
Geometrical model of the simulated unit

To check the response of the existing industrial CFB boiler at the changes of the operating conditions from air- to oxy-fuel mode, as well as influence of oxidizer composition on predicted temperature field the middle size compact CFB boiler was selected. This unit is operated in Poland at Turow Power Plant. Similarly as in early described CFB boiler the geometrical model of the CFB boiler was simplified to combustion chamber with all required gas and solid ports, similar as the earlier model. The geometrical model with marked inlets is depicted in Fig. 23 (left). This unit is equipped with four external solid separators located on the both boiler side walls. Unit is equipped with fuel and solid injection ports located on both boiler side walls. The secondary oxidizer gas is injected to combustion chamber through 26 ports distributed in three rows on trapped bed walls. In order to appropriately simulated material loop within the simplified boiler numerical mode the recirculation procedure was applied by set of proper user defined functions (UDFs) implemented into the solution procedure. The graphical illustration of the recirculation procedure is shown in Fig. 23 (right). The set of UDFs consist of several sub-procedure. First procedure is responsible for collecting information at the boiler outlet. This function removes particles which touch the outflow boundary of the computational domain. For each removed particles the information like: mass, diameter, density, composition, temperature, etc. are collected and storage in the memory. The second procedure calculates overall mass of the removed particles, and redistributes the collected particles over appropriate recirculation inlets. The operating of this procedure depends on the boiler construction, and for each boiler it has to be appropriately fitted. The temperature of recirculated material, as well as gases at the inlets was defined based on measured values collected during normal boiler operating. The last step of the recirculation procedure is injection of the material into the combustion chamber. Here only short description of the recirculation procedure was given, more information can be found in [24, 26].

Similarly as for Lagisza model the grid was modelled as flat surface where the primary gas was injected to combustion chamber through its whole bottom cross section. The boiler model were meshed using a combination of tetrahedral and hexahedral elements, where total number of grid elements was 600,000. The boiler input data is presented in Table 9. When the oxy-fuel combustion process is investigated the composition of the oxidizer mixture injected to combustion chamber plays crucial role on the behaviour of the combustion process and heat exchange within the combustion chamber [125]. The coal composition was collected in Table 10, as well as overall boiler thermal load, and amount of burned coal. For oxy-fuel combustion, in case of retrofitting existing CFB boiler it has to be assumed that the the amount of oxidizer used in combustion process should be the same as that used in boiler under air mode. The same amount of oxidizer for both investigated cases maintained similar hydrodynamic conditions within the combustion chamber, what has been mentioned earlier. In presented work the composition of oxidizer was calculated based on amount of recirculated flue gases. Calculated compositions and other input parameters for investigated case are collected in Table 9. The particle size distributions of the coal, sand, and ash for investigated boiler was modelled using Rosin–Rammler distribution, where the model parameters are shown in Table 11. At the boundry of the combustion chamber the same assuptions have been used as those used for modelling Lagisza CFB boiler. The temperature at the saturated water side was constant, and set to 357 °C.

**Table 9:** Input data for numerical model

	AIR	OXY 1	OXY 2
Oxidizer mass flow rate (kg/s)	274.5		
$\hbox {X}_{\mathrm {O_2,oxi}}$ (%)	20.92	28.81	28.19
$\hbox {X}_{\mathrm {CO_2,oxi}}$ (%)	0.00	61.69	54.86
$\hbox {X}_{\mathrm {H_2O,oxi}}$ (%)	0.00	2.30	8.24
$\hbox {X}_{\mathrm {N_2,oxi}}$ (%)	78.78	7.20	8.71
$\hbox {H}_2\hbox {O}$ reduction in rec. fuel gases (%)		70	30
Temp. of rec. material (°C)	800–900		
Oxidizer temp. (°C)	277		
Coal temp. (°C)	100

**Table 10:** Ultimate and proximate analysis of the lignite coal burned in Turow boiler

Proximate (%)		Ultimate (%)	
Ash	21.8	C	28.82
Water	40.5	H	2.32
Vol	22.3	S	0.31
Char	15.4	N	0.29
		O	5.96
Low heating value (LHV_AR_) (kJ/kg)		10,600	
Coal flow rate (kg/s)		∼52.6

**Table 11:** Material properties for fluidization process

	Coal	Inert
Density (kg/m^3^)	1300	1800
Mean diameter (μm)	325	330
Max diameter (μm)	2000	1000
Min diameter (μm)	50	50
Distribution parameter	1.25	1.35
Number of diameters	6	8

### Effect of Radiation

**Fig. 24:**
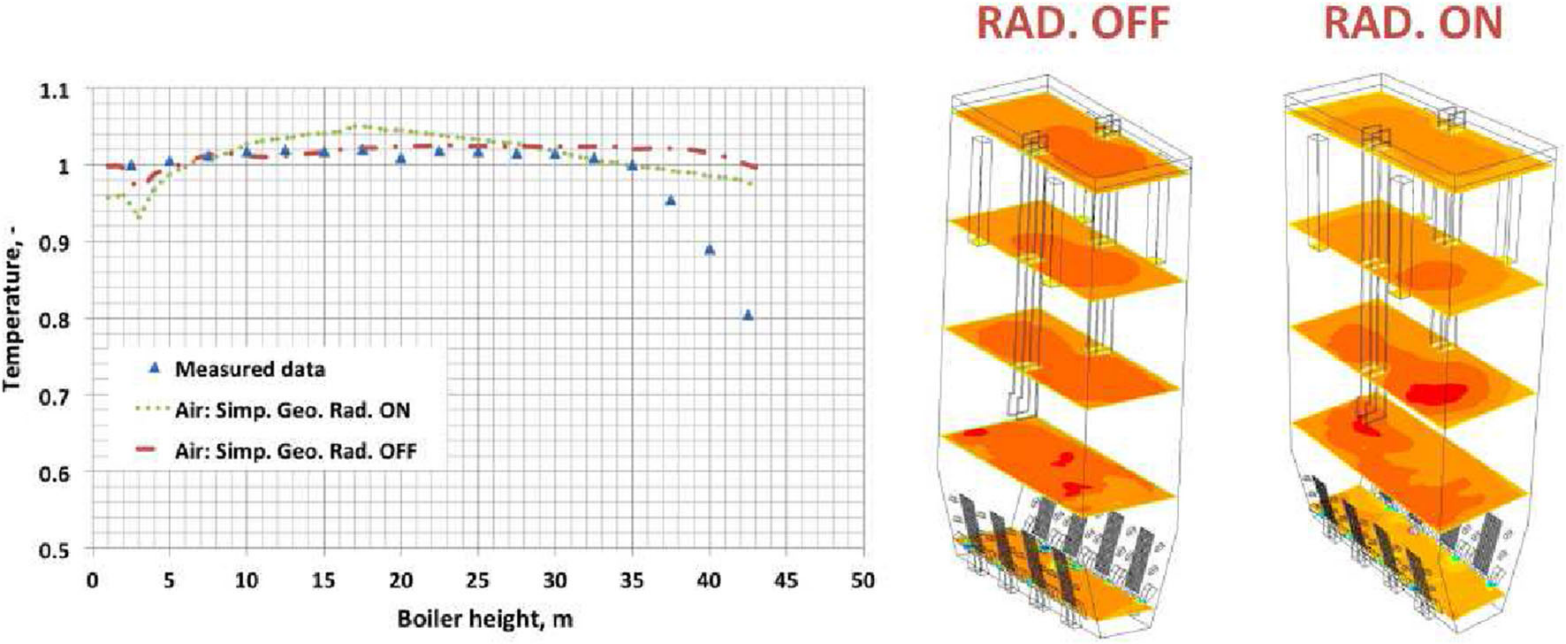
Calculated temperature profiles and distribution of temperature along combustion chamber with and without activated radiation model

The first set of simulations was performed in order to check the influence of radiation on evaluated temperature profile within the boiler. The parameters which mitigate the radiation effect within the CFB are presence of the high concentration of the particulate mater and relatively low combustion temperature. To confirm this hypothesis that the effect of radiation within the boiler can be safely neglected the air-combustion process was modelled with and without activated radiation model. The radiative heat transfer was modelled using standard Discrete Ordinate (DO) method. Calculated temperature profiles over boiler combustion chamber with and without activated radiation model are depicted in Fig. 24. Generally, the differences between the profiles are very small which confirms the hypothesis that radiation modelling within the CFB boiler can be safely neglected, which considerably reduces calculation time.

### Results of Air- and Oxy-Fuel Combustion

**Fig. 25:**
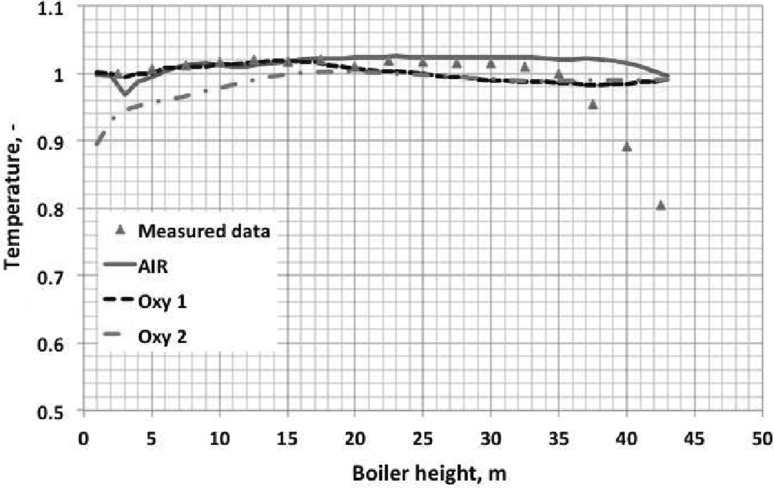
Scaled temperature profiles compared with measured data for *AIR* and two *OXY* cases

**Fig. 26:**
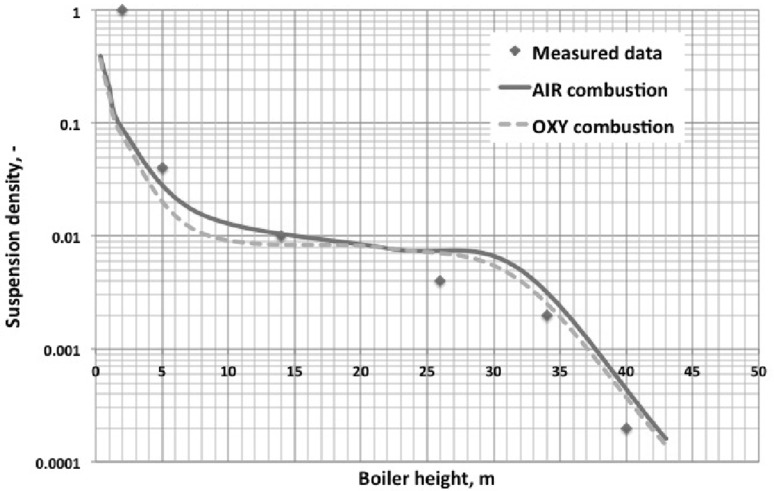
Measured and calculates suspension density over combustion chamber

**Fig. 27:**
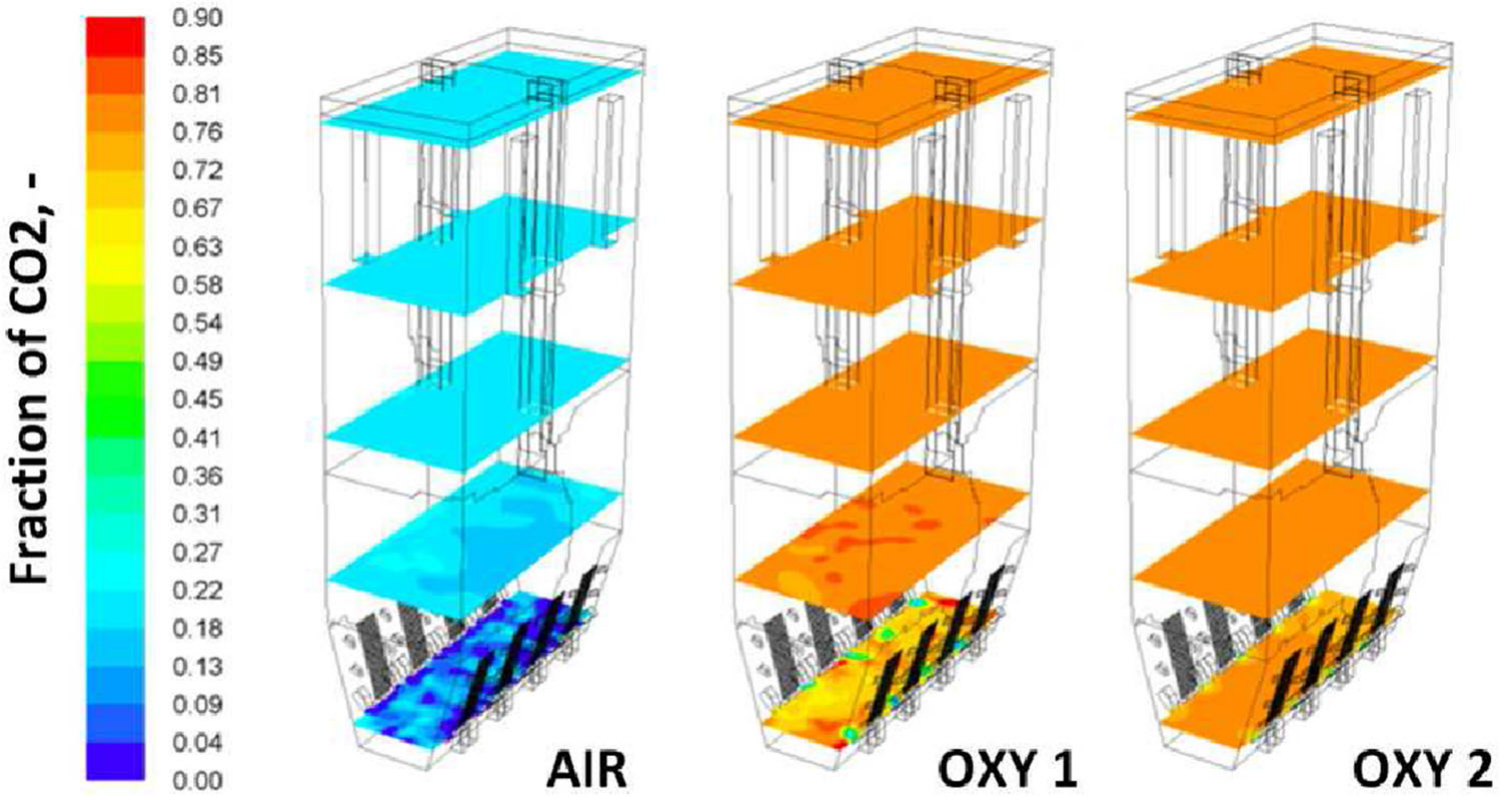
Distribution of carbon-dioxide over boiler combustion chamber for *AIR*, *OXY 1* and *OXY 2* cases

The temperature profiles calculated for the *AIR* and two *OXY* cases compared against measured data for boiler operated under air mode are illustrated in Fig. 25. The calculated profiles show comparable tendencies. Some discrepancies are observed at the bottom zone for case *OXY 2*. This situation was caused by increasing fraction of water in oxidizer composition, which decrease the temperature at this section. In Table 12 the average composition of the wet flue gases at the combustion chamber outlets for all investigated cases are collected. The distribution of suspension density over combustion chamber is shown in Fig. 26. Calculated and measured suspension density for *AIR* and *OXY 1* cases fit to each other. The difference between air- and oxy-fuel combustion can be very well observed base on the distribution of carbon-dioxide over the boiler combustion chamber which is depicted in Fig. 27. For oxy-fuel combustion, due to the recirculation of flue gases the fraction of $\hbox {CO}_2$ in flue gases is much higher in comparison to air-combustion which help in $\hbox {CO}_2$ septation and storage process.

**Table 12:** Flue gases composition for all investigated cases in wet molar basis

	AIR	OXY 1	OXY 2
$\hbox {X}_{\mathrm {O_2,fg}}$ (%)	2.7	5.8	2.71
$\hbox {X}_{\mathrm {CO_2,fg}}$ (%)	13.6	67.1	64.8
$\hbox {X}_{\mathrm {H_2O,fg}}$ (%)	14.6	21.6	25.7
$\hbox {X}_{\mathrm {N_2,fg}}$ (%)	69.1	5.5	6.8

## Conclusions

The main objective of the paper was to find, develop and test CFD model capable for solving particle transport and oxy-fuel combustion process in dens circulating fluidized bed (CFB) of a large scale industrial boiler. The fluidization process is in nature very complex and provides enormous problems during modelling. Although several known approaches can be used to deal with this complexities, no of earlier available numerical technique is seen as a robust and stable tool to simulate the CFB combustion process. Thus, the major aim of the work was to find the best available simulation technique, improve its stability, increase numerical efficiency and convergence. Though the core computer code used in the research was commercial Ansys FLUENT code, the underlying research required modification of the existing code via the user defined function (UDF) mechanism. Moreover, the application of the methods implemented in the Ansys FLUENT package was associated with selecting of proper combination of submodels, numerical techniques and careful control of the convergence process.

An important portion of the research underlying this paper was the experimental validation of the obtained numerical results. The philosophy behind the validation process was to begin with a simple model both in terms of geometry and physical phenomena and increase gradually the complexity of the simulation and experimental model in order not to introduce too many complexities at one step. Such an approach greatly simplified the diagnostics of the difficulties encountered when simulating the process.

At the first step of the validation process an *in-house* experimental facility was used to check the reliability of the simulation techniques and for the purpose of getting an insight into the hydrodynamics of the circulating fluidized bed. Special design of the test rig made it possible to use simple 2D geometry within the numerical simulation which considerable reduce computational effort. In next step the particle transport and oxy-fuel combustion process was simulated using a 3D model of small pilot test-rig built at Czestochowa University of Technology. The detailed description of the installation, numerical model and results were presented in earlier authors works [24, 25]. The gained experience on modeling of the combustion process in the two-dimensional rig and three-dimensional pilot scale rig, has been then extended to the large scale industrial CFB boilers.

The main problem encountered when developing the CFD model of the CFB is the large difference of geometrical and temporal scales in the transport phenomena occurring in the boiler modules (e.g., solid separator, drain section). To deal with these difficulties appropriate numerical model and solution method have to be used. The problem of large scale difference can be circumvented by development of simplified geometrical model of the CFB facility. Intuitive approach of predicting the particle transport is to apply the complete CFB geometry in order to reflect the complex nature of dense granular flow. However, long calculation times of such approach prevent from using this straightforward brute force approach. To reduce computational time, the complete CFB geometry has been replaced by the simplified CFB model, reducing the complete geometry to the combustion chamber only. The impact of model simplifications on the final results has been discussed in detail in other authors work [27].

The second portion of the research underlying this paper, a three dimensional model of a large scale industrial CFB boiler operated under air- and oxy-fuel combustion mode was presented. Moreover, due to the high influence of radiative active gases on the heat transfer process, mainly under oxy-fuel combustion an extended version of WSGG model presented in work [124] was implemented into the solution procedure. This model considerable increase simulation time, however in oxy-fuel mode standard WSGG model [113] cannot be used. Evaluated results for oxy-fuel combustion have shown some discrepancies in comparison to its air-fuel combustion case, mainly in vicinity to the secondary oxidizer inlets where the oxidizer velocity has been slightly modified due to other oxidizer composition. Performed simulations have shown that the CFB boiler retrofitting procedure was computationally expensive. Large difficulties were encountered during switching oxidizer from air- to oxy fuel mode due to large fluctuation of the temperature field. Additional instabilities with solution convergence were caused by used OXY WSGG model for spectrally active gases. Numerical simulations have shown that the numerical solution can be stabilize by performing the retrofitting procedure incrementally.

There is a number of unanswered questions and open problems in the context of the applied methods and their improvement. Some of the most important are listed below.

- The results obtained by applying hybrid Euler–Lagrange model, mainly the distribution of the material over the boiler which is directly connected with the pressure profile can by improved. Some problems can be encountered when to cores mesh is used. Basic model overpredicts the drag force for course meshes. For dense meshes, the simulation time considerably increases, making the approach not useful for simulating industrial application. However, implementation of the energy minimization multi-scale (EMMS) model [40, 41] should fixed this problem. The EMMS model works as the sub-grid scale model for effective inter-phase drag force, using an implicit cluster diameter expression for the predicted force.
- For the industrial boiler, the geometry has been reduced to combustion chamber with parts of oxidizer and solid injection ports. The recirculation of solid material has been substituted by a set of UDFs. The amount of solid material, for all investigated boiler loads has been calculated based on the measured pressure profiles [82]. To accurately predict the particle transport and mass distribution within the combustion chamber, the influence of external solid superheaters and solid separators should be taken into account. These sections should be included in simulations by extending the computational domain or development of a procedure in which, each of the boiler sections will be simulated separately and combined by specially written UDFs.
- The experience in the simulations gained so far suggests that there are difficulties in satisfying the mass conservation of the solid phase. This difficulty is common to all Lagrangian models. The problem can be resolved by applying the Eulerian model for combustion in the granular phase. The disadvantage of this approach is the usage of a number of somewhat artificial models and closure terms as well as long execution times when compared to the hybrid Euler–Lagrange technique.
- The calculation time should be reduced by developing pseudo steady-state CFD model for fluidization process.
- An important problem is the lack of kinetic models dedicated to burning coal in oxy-fuel combustion. The presented numerical results related to oxy-fuel combustion have been obtained using combustion models suitable for air-fuel combustion. The simple models neglect the influence of reach $\hbox {CO}_2$ atmosphere on diffusion process, which is important in kinetically controlled combustion regime. Appropriate models are currently in a development stage. Nowadays, to take into account the char gasification reaction during air- and oxy-fuel combustion process, more sophisticated, multi-surface reaction model have to be used. These models replace the simple kinetic/diffusion char reaction rate model. However, such an approach requires exact kinetic data for the burning of coal. The kinetic data should be obtained experimentally. The drop-tube reactor technique suitable for investigating the pulverized coal combustion kinetics, should not be used in fluidized bed context for two reasons: first due to large diameters of the fluidized bed coal particles, the height of the apparatus would be very large and second, the drop tube furnace experiment does not account for the strong particle interactions in the dense fluidized bed.
- The crucial parameters used in the sub-models of particle–wall and particle–particle collision processes is the collision and restitution coefficients, respectively. The only way it can be determined with reasonable accuracy is an experiment. The difficulty here is that the measurements should be performed using representative number of particles. Additional, difficulties are caused by un-spherical shape of the particle, whereas all numerical models assumed spherical particle shape.
- Fluidized boiler, due to presence of dense granular phase suffers to meany physical problems. The most import one is the erosion process which can caused serious problems in boiler operation. To improve boiler operating conditions, the regions in boiler exposed to high friction should be appropriately protected. To indicate such these regions numerical model can be used. Nevertheless, model presented in this work require several modification by implementation additional submodels to predict erosion and particle breakage process.

Future research will concentrate on:

- implementing the EMMS model for predicting drag force for coarse meshes,
- developing computation strategy for the industrial CFB boiler in order to reduce required time for numerical simulations,
- developing computation strategy which will be able to resolved particle transport within the combustion chamber, solid separators, downcomer, and drain section simultaneously in separate simulations, where numerical results will be linked between models by user defined functions,
- performing numerical simulations of the air- and oxy-fuel combustion process using the 3D experimental facility applying exact kinetic data for burned coal and extended boiler geometry encompassing solid separator and downcomer,
- improve radiation model for oxy-fuel combustion process, in order to decrease calculation time,
- application of the hybrid Euler–Lagrange approach for modeling particle transport, air- and oxy-fuel combustion processes within other large scale industrial CFB boilers,
- implementation submodels for predicting erosion process and particle breakage phenomena due to their collision.
